# EMG-driven musculoskeletal model calibration with estimation of unmeasured muscle excitations *via* synergy extrapolation

**DOI:** 10.3389/fbioe.2022.962959

**Published:** 2022-09-07

**Authors:** Di Ao, Marleny M. Vega, Mohammad S. Shourijeh, Carolynn Patten, Benjamin J. Fregly

**Affiliations:** ^1^ Rice Computational Neuromechanics Lab, Department of Mechanical Engineering, Rice University, Houston, TX, United States; ^2^ Biomechanics, Rehabilitation, and Integrative Neuroscience (BRaIN) Lab, VA Northern California Health Care System, Martinez, CA, United States; ^3^ Department of Physical Medicine and Rehabilitation, Davis School of Medicine, University of California, Sacramento, CA, United States

**Keywords:** muscle synergy, EMG-driven model calibration, synergy extrapolation, muscle excitation, stroke, muscle force

## Abstract

Subject-specific electromyography (EMG)-driven musculoskeletal models that predict muscle forces have the potential to enhance our knowledge of internal biomechanics and neural control of normal and pathological movements. However, technical gaps in experimental EMG measurement, such as inaccessibility of deep muscles using surface electrodes or an insufficient number of EMG channels, can cause difficulties in collecting EMG data from muscles that contribute substantially to joint moments, thereby hindering the ability of EMG-driven models to predict muscle forces and joint moments reliably. This study presents a novel computational approach to address the problem of a small number of missing EMG signals during EMG-driven model calibration. The approach (henceforth called “synergy extrapolation” or SynX) linearly combines time-varying synergy excitations extracted from measured muscle excitations to estimate 1) unmeasured muscle excitations and 2) residual muscle excitations added to measured muscle excitations. Time-invariant synergy vector weights defining the contribution of each measured synergy excitation to all unmeasured and residual muscle excitations were calibrated simultaneously with EMG-driven model parameters through a multi-objective optimization. The cost function was formulated as a trade-off between minimizing joint moment tracking errors and minimizing unmeasured and residual muscle activation magnitudes. We developed and evaluated the approach by treating a measured fine wire EMG signal (iliopsoas) as though it were “unmeasured” for walking datasets collected from two individuals post-stroke–one high functioning and one low functioning. How well unmeasured muscle excitations and activations could be predicted with SynX was assessed quantitatively for different combinations of SynX methodological choices, including the number of synergies and categories of variability in unmeasured and residual synergy vector weights across trials. The two best methodological combinations were identified, one for analyzing experimental walking trials used for calibration and another for analyzing experimental walking trials not used for calibration or for predicting new walking motions computationally. Both methodological combinations consistently provided reliable and efficient estimates of unmeasured muscle excitations and activations, muscle forces, and joint moments across both subjects. This approach broadens the possibilities for EMG-driven calibration of muscle-tendon properties in personalized neuromusculoskeletal models and may eventually contribute to the design of personalized treatments for mobility impairments.

## 1 Introduction

Muscle force is an important biomechanical variable for both research and clinical purposes. Knowledge of muscle forces during a variety of motion tasks could facilitate the development of more effective treatments for neuromusculoskeletal disorders such as stroke (Shao et al., 2009), Parkinson’s disease (Cano-de-la-Cuerda et al., 2010), and knee osteoarthritis (Kim et al., 2009). Specifically, such knowledge could lead to an improved understanding of the control strategies employed by the central neural systems (CNS) (Contessa and Luca, 2013; Del Vecchio et al., 2018), internal biomechanical quantities such as joint contact forces (Correa et al., 2010; Sasaki and Neptune, 2010; Manal and Buchanan, 2013; Walter et al., 2014; Serrancolí et al., 2016; Hoang et al., 2019), and external biomechanical quantities such as joint moments generated by muscles (Manal and Buchanan, 2003; Buchanan et al., 2005; Sartori et al., 2012; Meyer et al., 2017). While researchers are seeking to develop new experimental methods to measure muscle or tendon forces *in vivo* during human movement (e.g., Martin et al., 2018), direct measurement of muscle forces *in vivo* remains inherently challenging, costly, and ethically problematic to perform.

This situation has motivated the development of computational approaches that can estimate muscle forces from subject movement data. The two primary computational approaches used for this purpose are static optimization (SO) and EMG-driven musculoskeletal modeling. Both approaches typically utilize a geometric musculoskeletal model actuated by Hill-type muscle-tendon models (Zajac, 1989), where the control inputs to the muscle-tendon models can be either muscle excitations (equivalent to processed experimental-electromyographic (EMG) data) or muscle activations (muscle excitations after being passed through an activation dynamics model). SO is used to estimate muscle activations when EMG data are wholly (Crowninshield and Brand, 1981; Anderson and Pandy, 2001; Heintz and Gutierrez-Farewik, 2007) or partially (Sartori et al., 2014; Zonnino and Sergi, 2019) missing from important modeled muscles. This approach resolves the muscle redundancy problem by using nonlinear optimization to adjust the predicted muscle activations such that the sum of squares (or some other power) of muscle activations is minimized and the predicted net joint moments match the inverse dynamic net joint moments (Anderson and Pandy, 2001; Ackermann and van den Bogert, 2010). In contrast, EMG-driven modeling is used to estimate muscle excitations when EMG data are wholly or mostly available from important modeled muscles (Lloyd and Besier, 2003; Manal and Buchanan, 2003; Amarantini and Martin, 2004; Shao et al., 2009; Menegaldo et al., 2014; Sartori et al., 2014; Pizzolato et al., 2015; Meyer et al., 2017). To resolve the muscle redundancy problem, this approach uses processed EMG data to define the shapes of the predicted muscle excitations and then uses nonlinear optimization to adjust activation dynamics and Hill-type muscle-tendon model parameter values such that the sum of squares of errors between predicted and inverse dynamic net joint moments is minimized. While SO is extremely fast computationally, it can underestimate muscle activations since co-activation between agonist and antagonist muscles is minimized (Herzog and Binding, 1992), and it can produce unrealistic abrupt activation changes since the optimization process solves each time frame independently (Vilimek, 2007; Schellenberg et al., 2015). More importantly, it does not provide a way to calibrate activation dynamics and muscle-tendon model parameter values to a subject’s movement data, which can adversely affect the reliability of the estimated muscle forces (Serrancolí et al., 2016).

While calibration of activation dynamics and muscle-tendon model parameter values is built into the EMG-driven modeling process, several practical challenges exist with collecting EMG data from all muscles that contribute significantly to a specified movement task (Sartori et al., 2014; Péter et al., 2019; Zonnino and Sergi, 2019; Ao et al., 2020; Gurchiek et al., 2020). First, surface electrodes, which are non-invasive and easily applied, cannot measure EMG signals from deep muscles that contribute significantly to joint moments. Common examples are the iliacus and psoas muscles (Sartori et al., 2014), which significantly contribute to the hip flexion moment during walking. Second, though fine wire electrodes can measure EMG signals from deep muscles, they are invasive, require special skill and significant preparation time to insert, and may cause the subject discomfort and pain, limiting their utilization. Third, in some situations, deep muscles may not be reachable by any type of electrode. For example, use of a fine wire electrode may be contraindicated for safety reasons in subjects who have a cancerous tumor near an important deep muscle. Fourth, the number of available EMG channels is often less than that needed to drive a neuromusculoskeletal model for multi-joint movements (e.g., typically more than 10 channels per leg for walking and running). However, human movement labs often have only eight or 16 channels available for EMG recording. These challenges are important since missing EMG data from critical muscles may have a domino effect on the reliability of force estimates for other muscles that span the same joints (Pizzolato et al., 2015; Zonnino and Sergi, 2019).

Given the challenges described above, researchers have sought to develop various computational methods for estimating missing EMG signals during the EMG-driven model calibration process. One such method utilizes Gaussian process regression models to describe the synergistic relationship between a subset of muscles, enabling the estimation of unmeasured muscle excitations using information provided by a subset of measured muscle excitations (Gurchiek et al., 2020). However, muscle excitations associated with “unmeasured” muscles must be known initially to perform the necessary model training process, making this method infeasible when the “unmeasured” muscle excitations are truly unmeasurable due to experimental limitations or safety considerations. Another method utilizes low-dimension a sets of impulsive excitation primitives to estimate unmeasured muscle excitations (Neptune et al., 2009; Sartori et al., 2013; Pizzolato et al., 2015). Once excitation primitives are derived from measured muscle excitations, each muscle is assigned to a module by assessing associated weighting factors, where muscles without EMG measurements are assumed to belong to the same module as measured muscles that share the same innervation and contribute to the same mechanical action. Pizzolato et al. (2015) also minimally adjusted primitive-driven excitaitons for muscles with experimental EMG data to improve joint moment estimation in EMG-assisted mode. However, these adjustments masked the omission of active force generating properties for muscles without EMG data (i.e., iliacus and psoas), resulting in noticible hip joint moment prediction errors. Furthermore, none of these studies evaluated the accuracy of predicted umeasurd muscle excitations due to the lack of corresponding experimental EMG data.

More recently, the muscle synergy concept has been investigated for estimating muscle activations via SO or muscle excitations via EMG-driven modeling (Bianco et al., 2018; Ao et al., 2020; Michaud et al., 2020; Shourijeh and Fregly, 2020). A muscle synergy consists of a time-varying synergy excitation (or activation) and a corresponding time-invariant synergy vector containing weights that define how the synergy excitation (or activation) contributes to the excitation (or activation) of all muscles (Cappellini et al., 2006; Tresch et al., 2006; Ting and Chvatal, 2010; Banks et al., 2017). Muscle synergies are useful because they allow a large number of measured or modeled muscle excitations (or activations) to be represented by a small number of muscle synergies (typically between 3 and 6) (Tresch et al., 2006; Ivanenko et al., 2005; Ting and Chvatal, 2010; Banks et al., 2017). Michaud et al. (2020) and Shourijeh and Fregly (2020) imposed a synergy structure on muscle activations estimated via SO, which required solving for muscle activations over all time frames simultaneously. In both studies, muscle-tendon model parameter values were not calibrated simultaneously, no experimental EMG data were used to inform the muscle activation solutions, and the accuracy of predicted muscle activations compared to experimental EMG measurements were no better than from SO. In another recent study, Ao et al. (2020) used synergy excitations calculated from measured muscle excitations to predict synergy vector weights for unmeasured muscle excitations via a simplied EMG-driven modeling process. To evaluate the feasibility of the approach (which was termed “synergy extrapolation” or SynX), the authors used an EMG-driven model whose activation dynamics and muscle-tendon model parameters were already calibrated to subject movement data using a complete set of EMG measurements. Predictions of muscle excitations that were measured experimentally using fine wire electrodes but treated as unmeasured for SynX evaluation purposes showed excellent agreement with corresponding EMG measurements. However, since a pre-calibrated EMG-driven model was used for the evaluation, it remains unknown whether SynX can predict unmeasured muscle excitations reliably when EMG-driven model calibration is performed simultaneously.

This study extends the capabilities of SynX so that it can estimate missing EMG signals while simultaneously calibrating activation dynamics and muscle-tendon model parameter values in an EMG-driven model. The approach was developed and evaluated using gait datasets collected from two subjects post-stroke performing treadmill walking at self-selected and fastest-comfortable speeds. EMG signals measured bilaterally from iliopsoas using fine-wire electrodes were treated as “unmeasured” and used to evaluate the reliability of the method quantitatively. The computational approach uses nonlinear optimization to calibrate three categories of design variables simultaneously: 1) EMG-driven model parameters, 2) synergy vector weights and average values for constructing unmeasured excitations for muscles without associated EMG data, and 3) synergy vector weights and average values for constructing residual excitations for muscles with associated EMG data. The cost function was formulated as a trade-off between joint moment matching accuracy and unmeasured and residual muscle activation minimization. Different methodological choices, including the number of synergies and variability of synergy vector weights across trials, were investigated to determine the best choices for analyzing experimentally measured walking motions and generating computationally predicted walking motions. EMG-driven lower extremity models were calibrated for both subjects using the standard method with no missing EMG signals and SynX with missing iliacus and psoas EMG signals. Muscle excitations, activations, forces, and net joint moments along with EMG-driven model parameter values produced by the two calibration methods were compared for the walking trials used in the calibration process and additional walking trials held back for validation purposes.

## 2 Methods

### 2.1 Experimental data collection and processing

Previously published walking datasets collected from a high-functioning hemiparetic subject (S1, male, height 1.70 m, mass 80.5 kg, age 79 years, right-hand hemiparesis, lower extremity Fugl-Meyer Motor Assessment score of 32 out of a maximum 34) and a low-functioning hemiparetic subject post-stroke (S2, male, height 1.83 m, mass 88.5 kg, age 62 years, right-hand hemiparesis, lower extremity Fugl-Meyer Motor Assessment score of 25 out of a maximum 34) were used for this study (Meyer et al., 2017; Li et al., 2020). Video motion capture (Vicon Corp., Oxford, United Kingdom), ground reaction (Bertec Corp., Columbus, OH, United States), and EMG (Motion Lab Systems, Baton Rouge, LA, United States) data were recorded simultaneously while subjects walked on a split-belt instrumented treadmill (Bertec Corp., Columbus, OH, United States) at their self-selected (0.5 m/s for S1 and 0.45 m/s for S2) and fastest-comfortable (0.8 m/s for S1 and 0.65 m/s for S2) speeds. EMG data consisted of sixteen channels collected from each leg at 1,000 Hz using a combination of surface and fine wire electrodes (Table 1), which made EMG data available for important deep muscles such as iliopsoas. All experimental procedures were approved by the University of Florida Health Science Center Institutional Review Board (IRB-01), and both subjects provided written informed consent before participation. Raw motion capture and ground reaction data were low-pass filtered with a cut-off frequency of 7/*tf* Hz, where *tf* is the period of the gait cycle, while raw EMG data were high-pass filtered at 40 Hz, demeaned, full-wave rectified, low-pass filtered at 3.5/*tf* Hz, and normalized to maximum values over all experimental gait cycles (McLean et al., 2005; Meyer et al., 2017). Henceforth processed EMG data will be referred to as “muscle excitations.” Thirty-four and thirty-three muscles in each leg of the model that had either surface or fine-wire EMG data were kept for analysis for S1 and S2, respectively (Table 1). Data from ten gait cycles (five cycles per speed) per leg were randomly selected for EMG-driven model calibration. After pre-processing, data from each gait cycle were resampled to 101-time points representing initial heel-strike (0%) to subsequent heel-strike (100%) of the same foot. Twenty additional time frames before the start of each gait cycle were retained to account for a maximum electromechanical delay of approximately 100 ms for each muscle, which made each gait cycle possess 121 time points.

**TABLE 1:** List of muscles in the model for each subject and which DOF each muscle actuates.

Muscle names	Abbreviation	DOFs	Subject
Adductor brevis^a^	addbrev	HipFE/AA/Rot	Both
Adductor longus^a^	addlong		
Adductor magnus distal^a^	addmagDist		
Adductor magnus ischial^a^	addmagIsch		
Adductor magnus middle^a^	addmagMid		
Adductor magnus proximal^a^	addmagProx		
Gluteus maximus superior	glmax1		
Gluteus maximus middle	glmax2		
Gluteus maximus inferior	glmax3		
Gluteus medius anterior	glmed1		
Gluteus medius middle	glmed2		
Gluteus medius posterior	glmed3		
Gluteus minimus anterior	glmin1		
Gluteus minimus middle	glmin2		
Gluteus minimus posterior	glmin3		
Iliacus^a^	iliacus		
Psoas^a^	psoas		
Semimembranosus	semimem	HipFE/AA/Rot KneeFE	
Semitendinosus	semiten		
Rectus femoris	recfem		
Biceps femoris long head	bflh		
Tensor fasciae latae^a^	tfl		S2
Biceps femoris short head	bfsh	KneeFE	Both
Vastus medialis	vasmed		
Vastus intermedius	vasint		
Vastus lateralis	vaslat		
Lateral gastrocnemius	gaslat	KneeFE AnklePD/IE	
Medial gastronemius	gasmed	AnklePD/IE	
Tibialis anterior	tibant		
Tibialis posterior^a^	tibpost		
Peroneus brevis	perbrev		
Peroneus longus	perlong		
Soleus	soleus		
Extensor digitorum longus^a^	edl		S1
Flexor digitorum longus^a^	fdl		

a Measured using fine-wire EMG, electrodes.

### 2.2 Musculoskeletal model analyses

In preparation for EMG-driven model calibration, we performed a sequence of five musculoskeletal model analyses. First, a generic full-body OpenSim musculoskeletal model (Rajagopal et al., 2016) was scaled to match each subject’s anthropometry using OpenSim 4.0 (Delp et al., 2007; Seth et al., 2018). Each leg of the model possessed six degrees of freedom (DOFs), including hip flexion/extension (HipFE), hip adduction/abduction (HipAA), hip internal/external rotation (HipRot), knee flexion/extension (KneeFE), ankle plantarflexion/dorsiflexion (AnklePD), and ankle inversion/eversion (AnkleIE). Second, the locations and orientations of joint centers and axes, respectively, for the hip, knee, and ankle of each leg were adjusted via nonlinear optimization such that surface markers on the OpenSim model tracked experimentally measured surface marker positions as closely as possible for isolated joint motion and walking trials (Reinbolt et al., 2005). Third, inverse kinematic (IK) analyses were performed with OpenSim using experimental marker motion data from the walking trials to obtain the time histories of lower body joint angles. Fourth, inverse dynamic (ID) analyses were performed with OpenSim using IK joint angles and experimental ground reaction data for the same walking trials to calculate lower extremity joint moments. Fifth, for each muscle-tendon actuator in the model, a set of surrogate musculoskeletal geometric models was fitted to approximate the subject’s muscle-tendon lengths, velocities, and moment arms as a function of lower extremity joint angles and angular velocities (Menegaldo et al., 2004; Meyer et al., 2017).

### 2.3 EMG-driven model calibration with SynX

**FIGURE 1:**
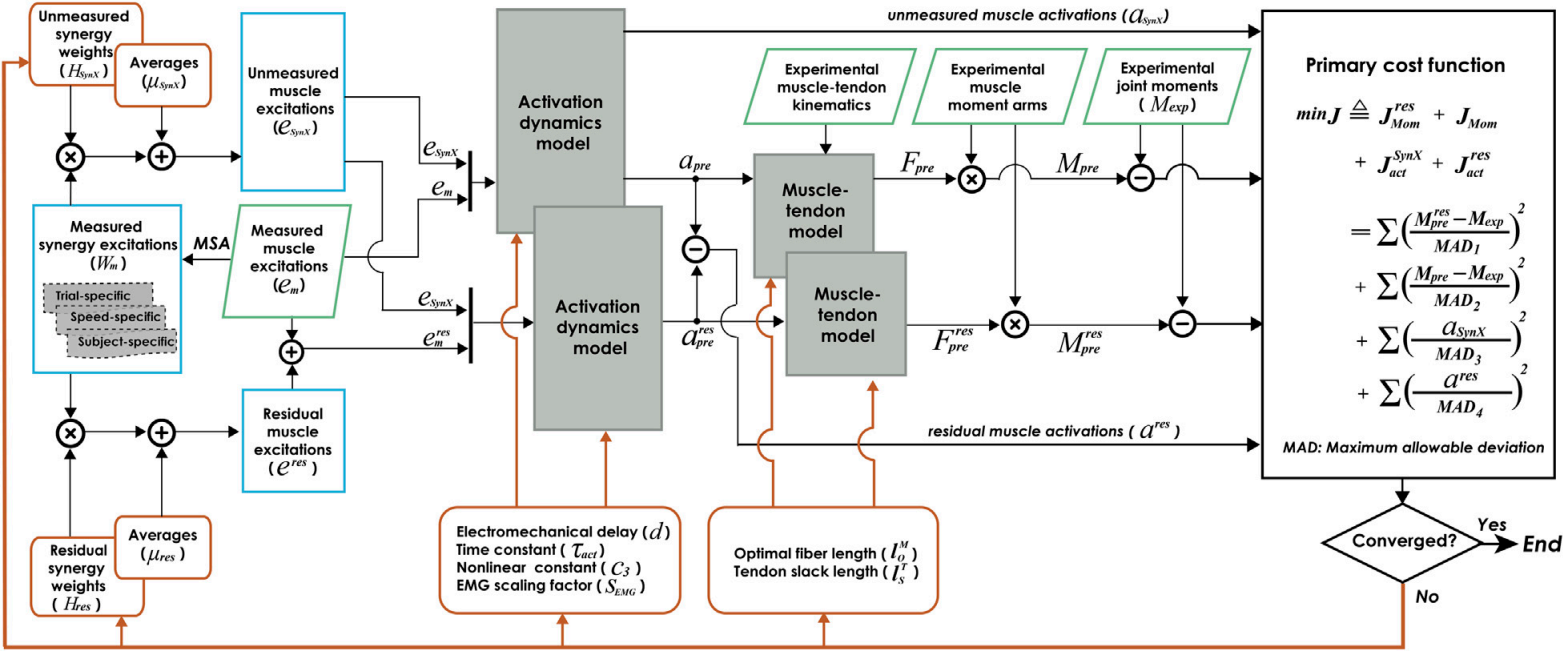
Flow chart of EMG-driven model calibration with synergy extrapolation (SynX) to estimating missing EMG signals. Orange rectangle boxes indicate optimization design variables, including activation dynamics model parameters, muscle-tendon model parameters, average values and synergy vector weights for unmeasured muscle excitations, and average values and synergy vector weights for residual excitations. The design variables were calibrated simultaneously by solving a nonlinear multi-objective optimization problem, described as “Params + SynX + Res” calibration. The objective function was formulated as a trade-off between joint moment tracking accuracy (with and without residual excitations included) and the magnitude of estimated unmeasured and residual muscle activations. Green parallelogram boxes indicate variables that were measured experimentally. Blue rectangle boxes indicate important intermediate variables generated within the SynX process. MSA stands for muscle synergy analysis.

The proposed computational method employing synergy extrapolation (SynX) to estimate missing muscle excitations during EMG-driven model calibration involves five steps (Figure 1), where all steps except the first one are performed within a nonlinear optimization process. First, muscle synergy analysis (MSA) is performed on measured muscle excitations via principal component analysis (PCA) to extract time-varying synergy excitations (henceforth referred to as “measured synergy excitations”) along with time-invariant synergy vector weights that define how each measured synergy excitation contributes to all measured muscle excitations (see details in *Extraction of measured synergy excitations*). Second, unmeasured muscle excitations are constructed by linearly combining the measured synergy excitations using the current guesses for the unmeasured synergy vector weights and average values (see details in *Estimation of unmeasured muscle excitations*). Third, residual muscle excitations added to the experimental muscle excitations are constructed by linearly combining measured synergy excitations using the current guesses for the residual synergy vector weights and average values (see details in *Estimation of residual muscle excitations*). Fourth, muscle activations, forces, and net joint moments are calculated by the EMG-driven musculoskeletal modeling using experimental joint kinematics and the current guesses for muscle excitations and muscle-tendon model parameter values when residual excitations are and are not included in the calculation of net joint moments (see details in *Formulation of EMG-driven musculoskeletal model*). Fifth, the nonlinear optimization adjusts all synergy and muscle-tendon model parameters to reduce the multi-objective cost function (see details in *Calibration of EMG-driven musculoskeletal model*). Below we describe each of these five steps as applied to 10 cycles of gait data for each leg of the two experimental subjects.

#### 2.3.1 Extraction of measured synergy excitations

Measured synergy excitations were extracted from the measured muscle excitations (excluding iliopsoas excitations when using SynX) by performing muscle synergy analysis (MSA). A previous study explored how methodological choices involved in MSA affect synergy extrapolation performance when a pre-calibrated EMG-driven musculoskeletal model is used (Ao et al., 2020). That study demonstrated that principal component analysis (PCA) with five or six synergies consistently predicted unmeasured muscle excitations accurately, while non-negative matrix factorization (NMF) could not achieve acceptable prediction accuracy. Moreover, EMG normalization did not significantly affect synergy extrapolation performance. Thus, in this study, measured muscle excitations were normalized to their maximum values over all trials, and PCA was selected for performing MSA. For between four and seven synergies, measured muscle excitations were decomposed via PCA and represented as:

$$e_{m}=\;W_{m}H_{m}+\mu_{m}+\varepsilon_{m}$$ (1)

where $e_{m}$ stands for measured muscle excitations, $W_{m}$ for time-varying measured synergy excitations, and $H_{m}$ for associated measured synergy vector weights. $\varepsilon_{m}$ accounts for the decomposition residuals that could not be explained by $W_{m}H_{m}+\mu_{m}$ , where $\mu_{m}$ specifies the average value of each measured muscle excitation.

MSA was performed to extract measured synergy excitations based on three assumptions about how measured synergy vector weights vary across trials. First, measured synergy vector weights were assumed to be trial-specific, necessitating a separate PCA decomposition for each trial individually, where $e_{m}$ was a *n*_*trial*_ (frames per trial) × *k* (measured excitations) matrix. *W*_*m*_ became an *n*_*trial*_ (frames per trial) × *p* (synergies) matrix and $H_{m}$ became a = *p* (synergies) × *k* (measured excitations) matrix. Second, measured synergy vector weights were assumed to be speed-specific, where the measured muscle excitations from trials with the same speed were concatenated such that $e_{m}$ became *n*_*speed*_ (frames per speed) × *k* (measured excitations). Thus, $W_{m}$ became *n*_*speed*_ (frames per speed) × *p* (synergies) and $H_{m}$ became p (synergies) × *k* (measured excitations). Third, measured synergy vector weights were assumed to be subject-specific. For this assumption, PCA was run on a concatenated matrix of measured muscle excitations over all trials, where $\;e_{m}$ was *n*_*subject*_ (frames over all trials) by k (measured excitations). $W_{m}$ had dimension of *n*_*subject*_ (frames over all trials) × *p* (synergies) and $H_{m}$ had the dimension of *p* (synergies) × *k* (measured excitations). In this study, five trials were analyzed for each of the two walking speeds, which led *n*_*speed*_ to be 605 [121 (frames per trial) × 5 (trials per speed)] and *n*_*subject*_ to be 1,210 [121 (frames per trial) × 10 (trials over two speeds)]. PCA-based MSAs were performed using the “pca” command in MATLAB (The Mathworks, Natick, MA).

#### 2.3.2 Estimation of unmeasured muscle excitations

Following MSA, unmeasured muscle excitations were constructed from the measured synergy excitations $W_{m}$ as shown in Eq. 2 below:

$$e_{SynX}=\;W_{m}H_{SynX}+\mu_{SynX}$$ (2)

where $e_{SynX}$ represents the unmeasured muscle excitations, $H_{SynX}$ represents the unmeasured synergy vector weights, and $\mu_{SynX}$ represents the average value of each unmeasured muscle excitation. Both $H_{SynX}$ and $\mu_{SynX}$ were design variables in the optimization problems formulated within our EMG-driven calibration approach (see more details in Section 2.3.5). Unmeasured synergy vector weights were assumed to be trial-specific, speed-specific, or subject-specific (henceforth referred to as “categories of unmeasured synergy vector weights”), where $W_{m}\;$ used for reconstruction was consistent with the corresponding category. More specifically, ten sets of $H_{SynX}$ and $\mu_{SynX}$ were generated for the trial-specific category, two sets of $H_{SynX}\;$ and $\mu_{SynX}$ for the speed-specific category, and only one set of $H_{SynX}$ and $\mu_{SynX}$ for the subject-specific category. For all categories of unmeasured synergy vector weights, $H_{SynX}$ was of dimensions *p* (synergies) × *q* (unmeasured excitations) and $\mu_{SynX}$ was 1 × *q* (unmeasured excitations).

#### 2.3.3 Estimation of residual muscle excitations

Similar to unmeasured muscle excitations, residual muscle excitations were constructed from the measured synergy excitations $W_{m}$ using the following relationship:

$$e^{res}=\;W_{m}H_{res}+\mu_{res}$$ (3)

where $e^{res}$ denotes residual muscle excitations, $H_{res}$ denotes residual synergy vector weights, and $\mu_{res}$ denotes the average value of the residual muscle excitation. Again, residual synergy vector weights were assumed to be trial-specific, speed-specific, or subject-specific specific (henceforth referred to as “categories of residual synergy vector weights”), where $W_{m}\;$ used for reconstruction was consistent with the corresponding category. For all categories of residual synergy vector weights, $H_{res}$ was of dimensions *p* (synergies) × *q* (measured excitations) and $\mu_{res}$ was 1 × *q* (measured excitations). The estimated residual muscle excitations were added to the measured muscle excitations to produce adjusted measured excitations:

$$e_{m}^{res}=\;e_{m}+e^{res}$$ (4)

where $e_{m}^{res}$ refers to measured muscle excitations with residual excitations included.

#### 2.3.4 Formulation of EMG-driven musculoskeletal model

Once unmeasured and residual muscle excitations were constructed, an EMG-driven musculoskeletal model was used to predict the six lower extremity net joint moments (Lloyd and Besier, 2003; Manal and Buchanan, 2003; Amarantini and Martin, 2004; Shao et al., 2009; Menegaldo et al., 2014; Sartori et al., 2014; Pizzolato et al., 2015; Meyer et al., 2017; Ao et al., 2020). First, muscle excitations *e(t)* were scaled by muscle-specific scale factors $s_{EMG}$ between 0.05 and 1 to reflect the fact that true maximum excitation levels are likely to be higher than those observed experimentally. Next, neural activation u(t) was calculated from muscle excitation e(t) using a published activation dynamics model (He et al., 1991) that used a first-order ordinary differential equation to define the relationship between e(t) and u(t) :

$$\frac{du\left(t\right)}{dt}=\left(c_{1}e\left(t-d\right)+c_{2})(e\left(t-d\right)-u\left(t\right)\right)$$ (5)

$$c_{1}=1/\tau_{act}-1/\tau_{dact}\;$$ (6)

$$c_{2}=1/\tau_{dact}\;$$ (7)

where $\tau_{act}$ and $\tau_{dact}$ are activation and deactivation time constants, respectively, $\tau_{dact}$ was assumed to be $4\tau_{act}$ (Zajac, 1989; Meyer et al., 2017), and d denotes an electromechanical time delay. Once neural activation was computed, a nonlinear function was used to determine the corresponding muscle activation a(t) (Manal and Buchanan, 2003):

$$a\left(t\right)=\left(1-c_{3}\right)u\left(t\right)+c_{3}\left[\frac{g_{1}}{g_{2}{\left(u\left(t\right)+g_{3}\right)}^{g_{4}}+g_{5}}+1\right]$$ (8)

where $c_{3}$ is an activation nonlinearity constant, $g_{1}$ to $g_{5}$ are constant coefficients determined by fitting published experimental data from isometric contractions (Manal and Buchanan, 2003).

Two sets of muscle activations were calculated when residual muscle excitations were and were not included, respectively. The muscle activations predicted from a combination of unmeasured muscle excitations $\left(e_{SynX}\right)$ and measured muscle excitations $\left(e_{m}\right)$ were defined as $a_{pre}$ , while the muscle activations predicted from a combination of unmeasured muscle excitations ( $e_{SynX}$ ) and measured muscle excitations with residual excitations included $\left(e_{m}^{res}\right)$ were defined as $a_{pre}^{res}$ . Residual muscle activations $a_{res}$ were then defined as the difference between $a_{pre}^{res}$ and $a_{pre}$ :

$$a^{res}=\;a_{pre}^{res}-\;a_{pre}$$ (9)

Next, taking both $a_{pre}$ and $a_{pre}^{res}$ as inputs, a Hill-type muscle-tendon model with rigid tendon (Hill, 1938; Zajac, 1989; Meyer et al., 2017) was used to predict the force generated by a given muscle-tendon actuator as shown below:

$$F\left(t\right)=F_{o}^{M}\cdot \left[a\left(t\right)\cdot f_{l}\left({\tilde{l}}^{M}\left(t\right)\right)\cdot f_{v}\left({\tilde{v}}^{M}\left(t\right)\right)+f_{p}\left({\tilde{l}}^{M}\left(t\right)\right)\right]\cos\alpha$$ (10)

where *F(t)* is the force generated by the muscle-tendon actuator, $F_{o}^{M}$ is the maximum isometric force of the muscle, a is the muscle activation, ${\tilde{l}}^{M}\left(t\right)$ and ${\tilde{v}}^{M}\left(t\right)$ are the time-varying normalized muscle fiber length and velocity, respectively, and α is the pennation angle of the muscle. $f_{l}\left({\tilde{l}}^{M}\left(t\right)\right)$ and $f_{v}\left({\tilde{v}}^{M}\left(t\right)\right)$ define the normalized active muscle force-length and force-velocity relationships, respectively, while $f_{p}\left({\tilde{l}}^{M}\left(t\right)\right)$ defines the normalized passive muscle force-length relationship. ${\tilde{l}}^{M}\left(t\right)$ and ${\tilde{v}}^{M}\left(t\right)$ were calculated using the following equations, which assume a rigid tendon:

$${\tilde{l}}^{M}\left(t\right)=\frac{l^{MT}\left(t\right)-l_{s}^{T}}{l_{o}^{M}}$$ (11)

$${\tilde{v}}^{M}\left(t\right)=\frac{v^{MT}\left(t\right)}{10\cdot l_{o}^{M}}$$ (12)

where $l^{MT}$ is muscle-tendon length and $v^{MT}$ muscle-tendon velocity, $l_{o}^{M}$ is optimal muscle fiber length, and $l_{s}^{T}$ is tendon slack length. With this muscle-tendon model, muscle forces were estimated when residual excitations were included $\left(F_{pre}^{res}\right)$ and not included $\left(F_{pre}\right)$ during prediction respectively.

Once $F_{pre}^{res}$ and $F_{pre}$ were computed for all muscles in the model, their contributions to net joint moments M were calculated using the corresponding muscle moment arms:

M(t)=∑F(t)· r(t) (13)

where r is muscle moment arm defined as the negative of the partial derivative of muscle-tendon length $l^{MT}$ with respect to generalized coordinate θ (An et al., 1984):

$$r\left(t\right)=-\frac{\partial l^{MT}\left(t\right)}{\partial \theta }$$ (14)

The negative sign in Eq. 14 was implemented for consistency with the OpenSim modeling environment. As required by the cost function for EMG-driven model calibration, net joint moments were calculated when residual excitations were $\left(M_{pre}^{res}\right)$ and were not $\left(M_{pre}\right)$ included in the measured muscle excitations.

#### 2.3.5 Calibration of EMG-driven musculoskeletal model

Calibration of the EMG-driven musculoskeletal model with simultaneous estimation of missing iliacus and psoas EMG signals was performed by using nonlinear optimization to adjust four categories of design variables: 1) activation dynamics model parameters consisting of EMG scale factor, $s_{EMG}$ , electromechanical delay d , activation time constant $\tau_{act}$ , and activation nonlinearity constant $c_{3}$ , 2) scaling factors for muscle-tendon model parameters consisting of optimal muscle fiber length $l_{o}^{M}$ and tendon slack length $l_{s}^{T}$ , 3) synergy vector weights $H_{SynX}$ and average values $\mu_{SynX}$ associated with unmeasured muscle excitations, and 4) synergy vector weights $H_{res}$ and average values $\mu_{res}$ associated with residual muscle excitations. To develop and evaluate the performance of our SynX approach, we formulated three optimization problems using various combinations of design variables (Table 2). First, to estimate missing EMG signals, we calibrated EMG-driven model parameters, synergy vector weights plus average values for unmeasured muscle excitations, and synergy vector weights plus average values for residual muscle excitations (termed “Params + SynX + Res,” see details in *Case 1: Params* + *SynX* + *Res*). Second, to explore how SynX performance is influenced by the inclusion of residual muscle excitations, we calibrated only EMG-driven model parameters and synergy vectors weights plus average values for unmeasured muscle excitations (termed “Params + SynX,” see details in *Case 2: Params* + *SynX*). Third, to evaluate how well important biomechanical variables (e.g., muscle activations, muscle forces, and net joint moments) can be estimated using “Params + SynX + Res” calibration, we calibrated only EMG-driven model parameters using the complete set of EMG signals with no muscle excitations predicted by SynX (termed “Params,” see details in *Case 3: Params*). For all three optimization problems, if unmeasured or residual muscle excitations were needed, we explored all possible methodological combinations of the number of synergies, category of unmeasured synergy vector weights, and category of residual synergy vector weights.

**TABLE 2:** Summary of calibration cases, which were named based on categories of design variables included in the optimization problem formulation.

Abbreviation	EMG-driven model parameters		Unmeasured muscle excitations		Residual muscle excitations	
	Calibrated?	Values from?	Calibrated?	Values from?	Calibrated?	Values from?
Params + SynX + Res	Yes	—	Yes	—	Yes	—
Params + SynX	Yes	—	Yes	—	No	0
Params	Yes	—	No	Exp	No	0

Exp. means experimental; “Values from” column indicates which values were used if the variables were not calibrated through optimization.

##### 2.3.5.1 Case 1: Params + SynX + Res

EMG-driven model calibration typically adjusts muscle forces by altering muscle-tendon model parameter values such that the difference between model-predicted and inverse dynamic (ID) joint moments are minimized. However, when unmeasured muscle excitations are estimated via SynX during EMG-driven model calibration, four terms are minimized simultaneously: 1) sum of squares of errors between model-predicted $\left(M_{pre}^{res}\right)$ and inverse dynamic $\left(M_{exp}\right)$ joint moments when residual muscle excitations are included in joint moment calculations, termed $J_{Mom}^{res}$ ; 2) sum of squares of errors between model-predicted $\left(M_{pre}\right)$ and inverse dynamic $\left(M_{exp}\right)$ joint moments when residual muscle excitations are not included in the joint moment calculations, termed $J_{Mom}$ ; 3) sum of squares of unmeasured muscle activations estimated by SynX $\left(a_{SynX}\right)$ , termed $J_{act}^{SynX}$ ; and 4) sum of squares of residual muscle activations $\left(a^{res}\right)$ , termed $J_{act}^{res}$ . Thus, the cost function for EMG-driven model calibration with SynX was formulated as:

$$\min J≜J_{Mom}^{res}+J_{Mom}\;+J_{act}^{SynX}\;+J_{act}^{res}\;$$ (15)

where

$$J_{Mom}^{res}=\sum {\left(\frac{M_{pre}^{res}-M_{exp}}{MAD_{1}}\;\right)}^{2}$$ (16)

$$J_{Mom}=\sum {\left(\frac{M_{pre}-M_{exp}\;}{MAD_{2}}\right)}^{2}$$ (17)

$$J_{act}^{SynX}=\sum {\left(\frac{a_{SynX}}{MAD_{3}}\;\right)}^{2}$$ (18)

$$J_{act}^{res}=\sum {\left(\frac{a^{res}}{MAD_{4}}\;\right)}^{2}$$ (19)

All four cost function terms (16–19) were normalized by a maximum allowable deviation (MAD). A sensitivity analysis was performed to determine a MAD value for each cost function term, as described in the Appendix. Specific details about initial guesses and upper/lower bounds for each design variable, additional constraints, and cost function penalty terms can be found in Supplementary Table S1 in the Appendix and previously published papers (Meyer et al., 2017; Ao et al., 2020). All optimizations were performed using MATLAB’s “fmincon” function with the sequential quadratic programming algorithm.

##### 2.3.5.2 Case 2: Params + SynX

To assess how well unmeasured muscle excitations can be predicted when no residual muscle excitations are included, we estimated unmeasured muscle excitations as in Case 1 but without adding residual muscle excitations to the measured muscle excitations. For this case, the cost function was:

$$\min J≜\sum {\left(\frac{M_{pre}-M_{exp}\;}{MAD_{2}}\right)}^{2}\;+\sum {\left(\frac{a_{SynX}}{MAD_{3}}\;\right)}^{2}$$ (20)

where $M_{pre}$ denotes the joint moments calculated from a combination of measured $\left(e_{m}\right)$ and unmeasured $\left(e_{SynX}\right)$ muscle excitations.

##### 2.3.5.3 Case 3: Params

To evaluate the performance of our computational approach for different methodological choices, we performed EMG-driven model calibration using the full set of EMG signals, where no EMG signals were treated as “unmeasured,” and only activation dynamics and muscle-tendon model parameter values were optimized to match the experimental joint moments from inverse dynamics:

$$\min J≜\sum {\left(\frac{M_{pre}-M_{exp}}{MAD_{2}}\;\right)}^{2}$$ (21)

where $M_{pre}$ represents model-predicted net joint moments produced by a complete set of muscle excitations.

### 2.4 EMG-driven model evaluation

Several common metrics were used to evaluate the outcome of muscle synergy analysis and the reliability of unmeasured muscle excitations generated using SynX with all possible methodological combinations. First, the variance accounted for (VAF) was computed between experimental and reconstructed muscle excitations of measured muscles, where the number of synergies in each leg was determined using a threshold criterion of 95% VAF (Tresch et al., 2006; Steele et al., 2013). Next, the EMG-driven model calibration process utilizing SynX was evaluated in three stages. In the first stage, the influence of different methodological choices (i.e., number of synergies, category of unmeasured and residual synergy vector weights) on SynX performance was investigated, and the choices that produced the most reliable estimates of unmeasured muscle excitations were identified. The two “most reliable” SynX methodological combinations for two distinct situations were identified by quantifying how well unmeasured muscle excitations and activations for psoas and iliacus could be predicted. Root mean square error (RMSE) and Pearson correlation coefficient *r* between experimental (from “Params” case) and estimated (from “Params + SynX + Res” case) muscle excitations and activations for iliacus and psoas across two speeds were computed to quantify matching of magnitude and shape, respectively. Correlation was interpreted quantitatively as weak (r < 0.35), moderate (0.35 < r ≤ 0.67), strong (0.67 < r ≤ 0.9), or very strong (r ≤ 0.9) (Taylor, 1990). The first situation involved predictions made when synergy vector weights and average values for unmeasured muscle excitations, along with muscle-tendon model parameter values, were calibrated via SynX. This situation (called “calibration”) is how the EMG-driven model would analyze walking trials used in the calibration process, where synergy vector weights and average values for unmeasured and residual muscle excitations can be calibrated on a trial-specific, speed-specific, or subject-specific basis. The second situation involved predictions made when synergy vector weights and average values for unmeasured muscle excitations, along with muscle-tendon model parameter values, were pre-calibrated via SynX. This situation (called “validation”) is how the EMG-driven model would be used to analyze experimental walking trials not used in the calibration process or to generate computationally predicted walking motions, where pre-calibrated synergy vector weights and average values for unmeasured muscle excitations must be utilized on either a speed-specific or subject-specific basis (Meyer et al., 2016; Sauder et al., 2019). In the second stage, muscle activations, forces, and net joint moments from “Params + SynX + Res” calibration were compared with those from “Params” calibration for both subjects using the first “most reliable” methodological combination applied to 10 calibration walking trials (five per speed). In the third stage, muscle activations, forces, and net joint moments from “Params + SynX + Res” validation were compared with those from “Params” validation for both subjects using the second “most reliable” methodological combination applied to 10 validation walking trials (five per speed) not utilized in the calibration process. For this stage, unmeasured synergy vectors weights and average values were fixed at their calibrated values. Mean absolute errors (MAE) between inverse dynamic and model-predicted net joint moments were calculated for “Params + SynX + Res” and “Params” cases using calibration trials in the second stage and validation trials in the third stage, respectively. Variance accounted for (VAF) was computed between experimental and model-predicted unmeasured muscle excitations, where the number of synergies in each leg was determined using a threshold criterion of 95% VAF (Tresch et al., 2006; Steele et al., 2013).

Multiple statistical analyses were performed to assess whether the calculated metrics resulting from different SynX methodological choices were statistically different. To assess whether methodological choices for MSA had a statistically significant impact on the reconstruction performance of measured muscle excitations, we performed a two-factor ANOVA with a Tukey-Kramer post-hoc analysis on VAF values. Second, to compare SynX performance among different SynX methodological choices, we performed a three-factor ANOVA tests on *r* and RMSE values across both patients and all calibration trials, followed by paired *t*-tests for comparing categories of synergy vector weights for a given number of synergies. For example, when we compared the difference among categories of unmeasured synergy vector weights, we paired *r* or RMSE values such that each pair shared the same category of residual synergy vector weights and number of synergies when associated with the same leg. Third, to investigate whether the inclusion of residual muscle excitations influenced SynX performance, we performed paired *t*-tests on *r* and RMSE values between “Params + SynX + Res” calibration with each category of residual synergy vector weights and “Params + SynX” calibration with no residual excitations. Fourth, we conducted paired *t*-tests to compare the joint moment matching errors (MAE values) between “Params” calibration and “Params + SynX + Res” calibration, where residual excitations were used in the calibration process but not included in the final joint moment calculation. All statistical analyses were performed in MATLAB, and the level of statistical significance was set at *p* < 0.05.

## 3 Results

### 3.1 Analysis of experimental muscle synergies

The two-way ANOVA applied to mean VAF values between reconstructed and experimental muscle excitations of measured muscles revealed the main effects of the number of synergies (*p* < 0.05) and the category of measured synergy vector weights (*p* < 0.05) on the variance explained by the factorization of measured muscle excitations. Post-hoc analysis indicated that VAF values significantly increased as the number of synergies increased from four to seven (*p* < 0.05). Also, with the same number of synergies, trial-specific synergy vector weights provided the highest VAF values, while subject-specific synergy vector weights provided the lowest VAF values for both S1 and S2 (Table 3). Overall, when using trial-specific synergy vector weights, measured muscle excitations were predicted with >95% VAF with four synergies for S1 and S2. When synergy vector weights were shared within the same speed, six synergies were needed for the left leg of S1, seven synergies were needed for the right leg of S1 and the left leg of S2, and four synergies were needed for the right leg of S2. As synergy vector weights were held constant across all trials, seven synergies were required for all legs except for the right leg of S2, where only 5 synergies were needed.

**TABLE 3:** Mean ± standard deviation of VAF values for the reconstruction of measured muscle excitations with muscle synergy analysis across both subjects and all calibration trials with four to seven synergies when unmeasured synergy vector weights were assumed to be trial-specific, speed specific, or subject-specific.

Sub	Variability assumption	Left				Right			
		Number of synergies				Number of synergies			
		4	5	6	7	4	5	6	7
S1	Trial	**96.5 ± 1.5**	98.2 ± 0.7	99.3 ± 0.3	99.7 ± 0.1	**95.2 ± 2.4**	96.8 ± 1.8	98.6 ± 0.9	99.2 ± 0.5
	Speed	91.0 ± 3.6	93.7 ± 2.2	**96.1 ± 1.5**	97.4 ± 1.1	87.7 ± 5.6	92.2 ± 3.8	94.3 ± 2.5	**95.5 ± 2.0**
	Subject	89.2 ± 5.6	92.1 ± 3.3	94.8 ± 2.6	**96.2 ± 1.6**	84.4 ± 7.1	88.5 ± 3.6	92.2 ± 4.4	95.1 ± 3.3
S2	Trial	**95.3 ± 1.8**	97.6 ± 1.2	98.8 ± 0.6	99.5 ± 0.2	**97.3 ± 1.1**	98.6 ± 0.5	99.3 ± 0.3	99.6 ± 0.2
	Speed	89.3 ± 4.1	93.2 ± 2.6	93.2 ± 2.6	**97.1 ± 0.9**	**95.3 ± 0.9**	96.5 ± 0.9	97.3 ± 0.7	97.9 ± 0.4
	Subject	87.8 ± 4.5	91.3 ± 2.7	94.7 ± 1.9	**96.0 ± 1.7**	93.1 ± 1.4	**95.4 ± 0.8**	96.3 ± 0.7	97.1 ± 0.8

Sub. means subject bold represents the minimum number of synergies required for the specific leg to achieve a VAF above 95%.

### 3.2 First stage: Influence of methodological choices on SynX performance

**FIGURE 2:**
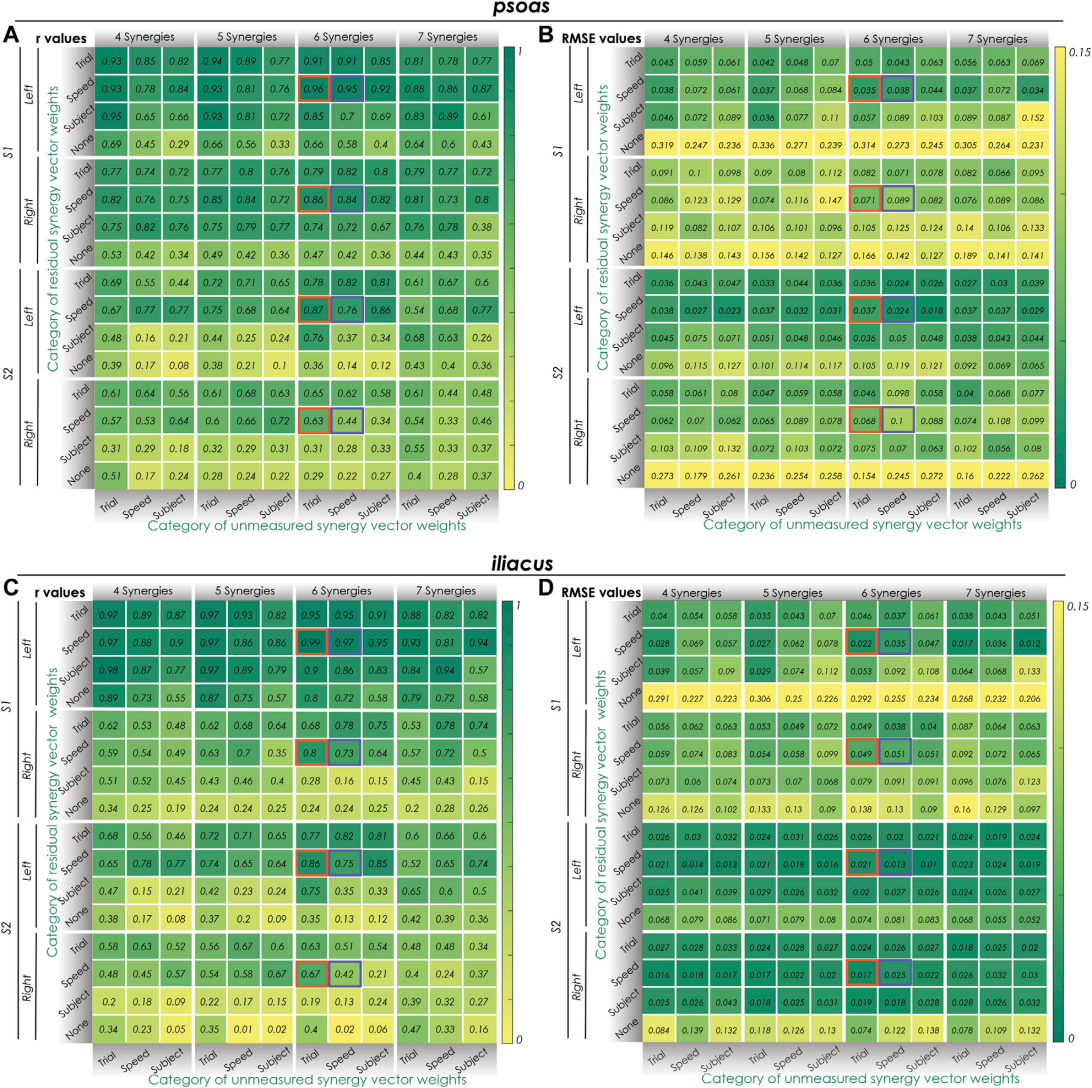
Synergy extrapolation performance for different methodological combinations when using the proposed EMG-driven calibration framework. **(A)** Pearson correlation coefficient r values and **(B)** root mean square error (RMSE) values for different methods of reconstructing psoas muscle activations across all calibration trials. **(C)** Pearson correlation coefficient r values and **(D)** root mean square error (RMSE) values for different methods of reconstructing iliacus muscle activations across all calibration trials. Unmeasured (bottom) and residual (side) synergy vector weights were categorized as either trial-specific, speed-specific, or subject-specific. Within the columns for 6 synergies, Orange boxes indicate the best SynX methodological combination (trial-specific unmeasured and speed-specific residual synergy vector weights) for calibration. The purple boxes indicate the best SynX methodological combination (speed-specific unmeasured and speed-specific residual synergy vector weights) for validation. Residual synergy vector weights categorized as “None” indicate calibration results for “Params + SynX” where no residual muscle excitations were predicted. These results suggest that “Params + SynX” calibration should be rejected due to unacceptable SynX performance.

In the first stage, the impact of different methodological combinations (i.e., number of synergies, category of unmeasured and residual synergy vector weights) on SynX performance was evaluated using r and RMSE values between predicted and measured iliopsoas muscle excitations. The three-factor ANOVA analyses revealed that the category of unmeasured synergy vector weights, category of residual synergy vector weights, and the number of synergies significantly affected both r and RMSE values that characterized SynX performance (*p* < 0.05). Several general observations were made by assessing the results for both psoas and iliacus and both subjects as a whole (Figure 2 and Supplementary Figure S2). First, without residual excitations being calibrated through optimization (labeled as “None” from “Params + SynX” calibration), r values were significantly lower, and RMSE values were significantly higher than those for any of the three categories of residual synergy vector weights (*p* < 0.05). Second, five and six synergies offered substantially higher r values and lower RMSE values than did four and seven synergies (*p* < 0.05), while no significant difference was detected between five and six synergies (*p* = 0.096). r values reached a maximum value, and RMSE values reached a minimum value at five synergies for the right leg of S2 or six synergies for both legs of S1 and the left leg of S2. Third, using speed-specific and trial-specific residual synergy vector weights yielded significantly better SynX performance than using subject-specific residual synergy vector weights (*p* < 0.05). Furthermore, when using five or six synergies, particularly trial-specific residual synergy vector weights, higher r and lower RMSE values were achieved when assuming them speed-specific. Fourth, with five or six synergies, using trial-specific synergy vector weights for the reconstruction of unmeasured muscle excitations provided the greatest r values and the lowest RMSE values, while subject-specific synergy vector weights provided the smallest r values and the biggest RMSE values (*p* < 0.05).

Two “most reliable” SynX methodological combinations were identified for “calibration” and “validation” situations, respectively. As highlighted by oranges boxes in Figure 2 and Supplementary Figure S2, best SynX performance for both unmeasured muscled and both subjects was provided with six synergies, trial-specific unmeasured synergy vector weights, and speed-specific residual synergy vector weights, which could be used to analyze walking trials used in the calibration process. Further results obtained in the second stage for the “calibration” situation were described in *Second stage: Evaluation of “calibration” situation* of the results. As indicated by purple boxes in Figure 2 and Supplementary Figure S2, to analyze experimental walking trials *not* used in the calibration process or to generate computationally predicted walking motions, a SynX-performing methodological combination was chosen using six synergies and speed-specific synergy vector weights for the reconstruction of both unmeasured muscle excitations and residual muscle excitations. Further results obtained in the third stage for the “validation” situation were described in *Third stage: Evaluation of “validation” situation* of the results.

### 3.3 Second stage: Evaluation of “calibration” situation

**FIGURE 3:**
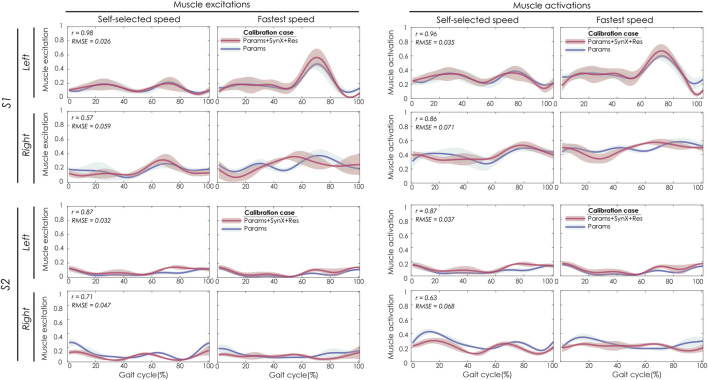
SynX-predicted muscle excitations and activations for psoas constructed with the best methodological combinations for the “calibration” situations (trial-specific unmeasured and speed-specific residual synergy vector weights with six synergies). Lines represent mean curves across calibration trials, and shaded areas represent ±1 standard deviation. r and RMSE values for muscle excitations and activations were calculated between “Params + SynX + Res” calibration (in pink) and “Params” calibration (in blue). “Params” calibration was performed using a complete set of EMG signals, where no muscle excitations were predicted. Data are reported for the complete gait cycle, where 0% indicates initial heel-strike and 100% indicates subsequent heel-strike for each leg of both subjects (right leg: paretic, left leg: nonparetic). SynX-predicted muscle excitations and activations for iliacus were of similar accuracy.

For “Params + SynX + Res” calibration, the most reliable estimates of unmeasured muscle excitations and activations were achieved using six synergies, trial-specific unmeasured synergy vector weights, and speed-specific residual synergy vector weights (Figure 3). Compared to the results from the “Params” calibration, unmeasured muscle excitations (left two columns) were predicted with strong correlations for the left leg of S1 and both legs of S2 and with moderate correlations for the right leg of S1. The corresponding unmeasured muscle activations (right two columns) were strongly correlated with those from “Params” calibrations for both legs of S1 and the left leg of S2 and moderately correlated for the right leg of S2. SynX-estimated muscle excitations and activations were predicted with low RMSE values (<0.071) across all legs.

**FIGURE 4:**
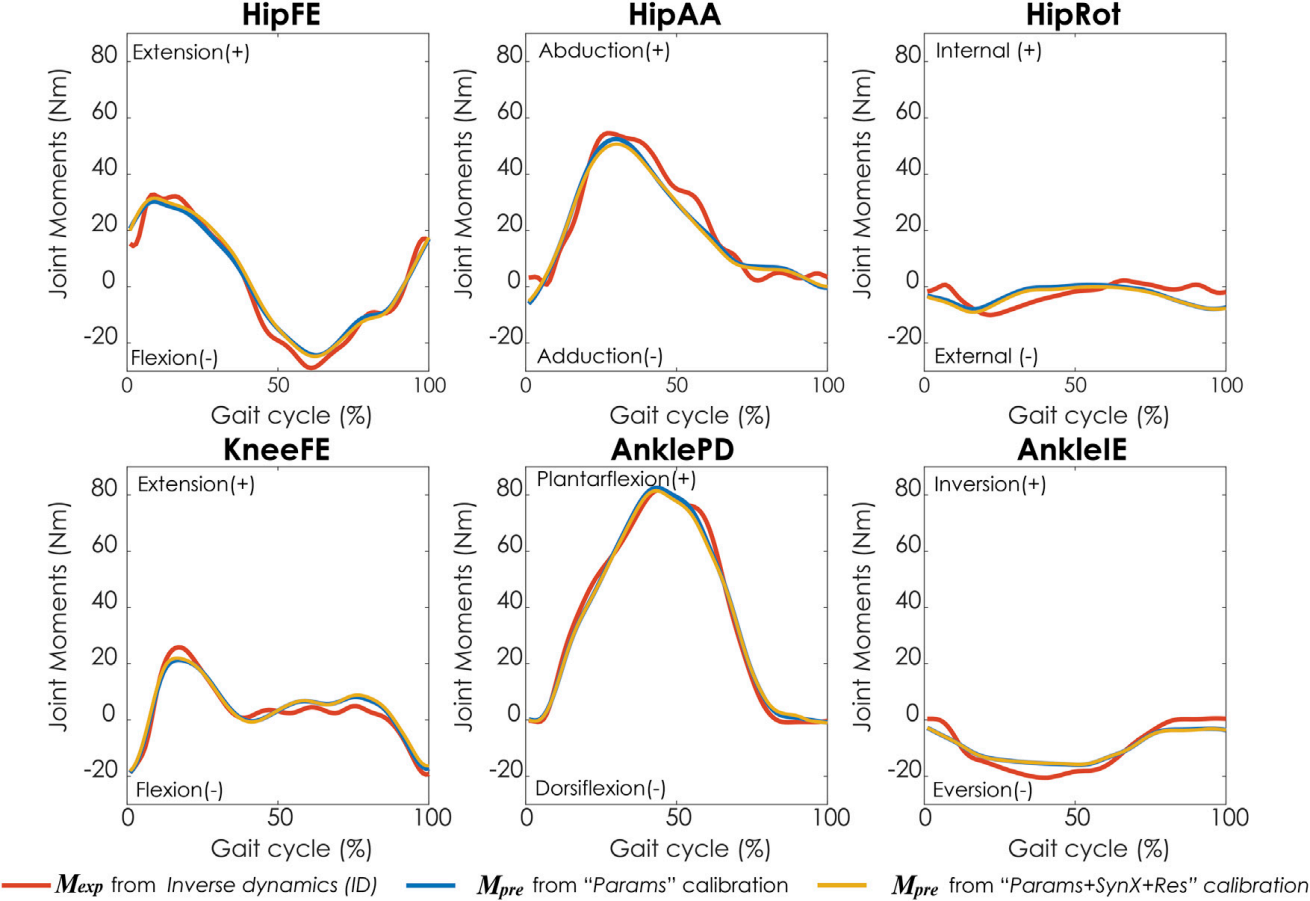
Average joint moments across calibration trials and subjects from inverse dynamics (in red), “Params” calibration (in blue), and “Params + SynX + Res” calibration (in yellow). The results for “Params + SynX + Res” calibration were generated with the best methodological combination for calibration conditions (trial-specific unmeasured and speed-specific residual synergy vector weights with 6 synergies). For “Params + SynX + Res,” residual excitations were calibrated to improve the prediction of unmeasured muscle excitations but not used to calculate joint moments. Data are reported for the complete gait cycle, where 0% indicates initial heel-strike and 100% indicates subsequent heel-strike.

**FIGURE 5:**
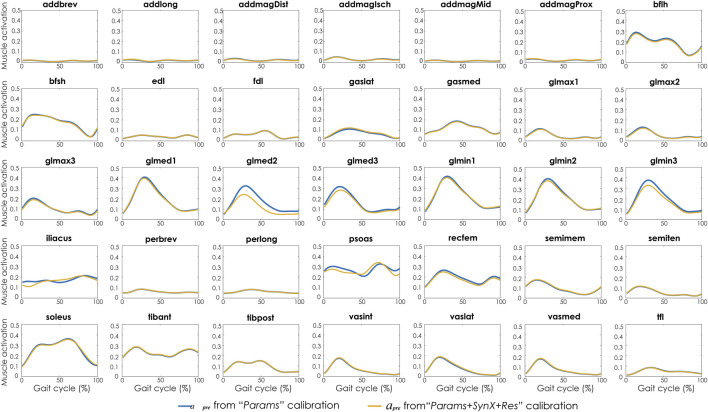
Average muscle activations across calibration trials and subjects from “Params” calibration (in blue), and “Params + SynX + Res” calibration (in yellow). Results for “Params + SynX + Res” calibration were generated using the best methodological combinations for analyzing experimentally measured walking motions (trial-specific unmeasured and speed-specific residual synergy vector weights with six synergies). Here, residual excitations were calibrated but not used to calculate muscle activations for “Params + SynX + Res.” Data are reported for the complete gait cycle, where 0% indicates initial heel-strike and 100% indicates subsequent heel-strike.

**FIGURE 6:**
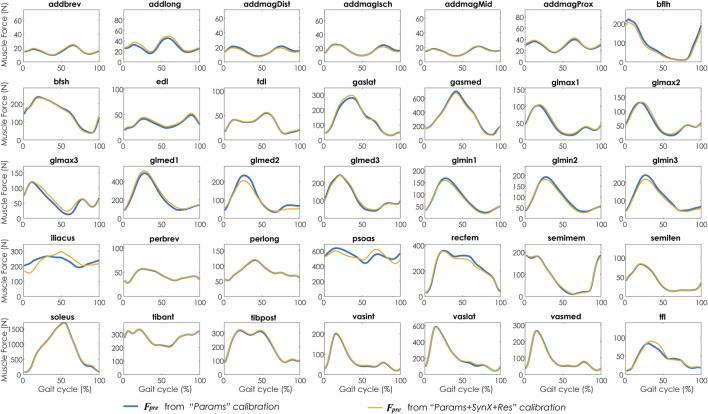
Average muscle forces across calibration trials and subjects from “Params” calibration (in blue) and “Params + SynX + Res” calibration (in yellow). Results for “Params + SynX + Res” calibration were produced using the best methodological combinations for analyzing experimentally measured walking motions (trial-specific unmeasured and speed-specific residual synergy vector weights with six synergies). Here, residual excitations were calibrated but not used to calculate muscle forces for “Params + SynX + Res.” Data are reported for the complete gait cycle, where 0% indicates initial heel-strike and 100% indicates subsequent heel-strike.

**FIGURE 7:**
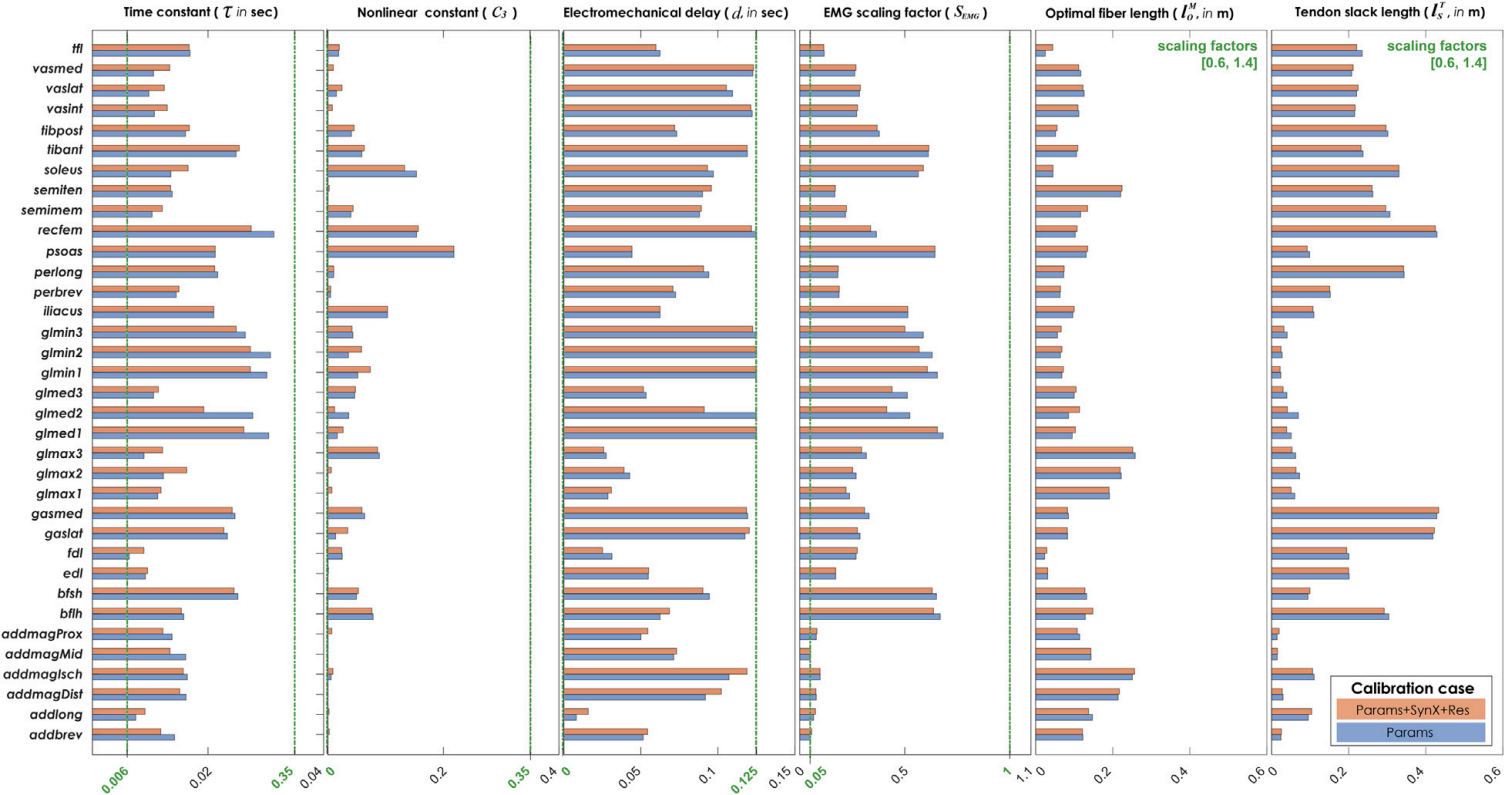
Average EMG-driven model parameters of both subjects from “Params” calibration (in blue) and “Params + SynX + Res” calibration (in orange) Results for “Params + SynX + Res” calibration were produced using the best performing methodological combinations for analyzing experimentally measured walking motions (trial-specific unmeasured and speed-specific residual synergy vector weights with six synergies). The upper and lower bounds for each of the four activation dynamics model parameters during optimization have been indicated by green vertical dash-sot lines, where the upper and lower bounds for the scaling factors of optimal fiber lengths and tendon slack lengths were [0.6, 1.4] for all muscles.

Compared with MAE values between model-predicted and experimental ID joint moments from “Params” calibrations, the MAE values were slightly lower but statistically comparable (*p* > 0.05) for all joints from “Params + SynX + Res” calibrations when residual excitations were calibrated but not used for joint moment calculation (Figure 4). However, the joint moment errors were significantly lowered (*p* < 0.05) when including residual muscle excitations in the prediction process (Supplementary Table S2 in the Appendix). On average, measured muscle activations generated from “Params + SynX + Res” calibration remained close to the those from “Params” calibration (Figure 5), where the greatest deviations were observed for the muscles that spanned the hip joint (e.g., glmed2). In addition, for measured and unmeasured muscles, muscle forces estimated from “Params + SynX + Res” calibration were in excellent agreement with those estimated from “Params” calibration in terms of both shape and magnitude (Figure 6). Moreover, in general, EMG-driven model parameter values were similar between “Params + SynX + Res” calibration and “Params” calibration (Figure 7). However, when additional variables (i.e., unmeasured muscle synergy vector weights and residual synergy vector weights) were tuned simultaneously, the pattern defined by the parameter magnitudes over all muscles was still retained for each model parameter.

### 3.4 Third stage: Evaluation of “validation” situation

**FIGURE 8:**
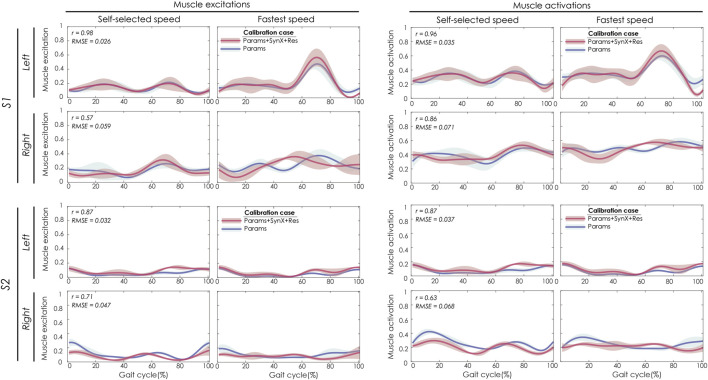
SynX-predicted muscle excitations and activations for psoas constructed with the best methodological combinations for the “validation” situations (speed-specific unmeasured and speed-specific residual synergy vector weights with six synergies). Lines represent mean curves across calibration trials, and shaded areas represent ±1 standard deviation. r and RMSE values for muscle excitations and activations were calculated between “Params + SynX + Res” calibration (in pink) and “Params” calibration (in blue). “Params” calibration was performed using a complete set of EMG signals, where no muscle excitations were predicted. Data are reported for the complete gait cycle, where 0% indicates initial heel-strike and 100% indicates subsequent heel-strike for each leg of both subjects (right leg: paretic, left leg: nonparetic). SynX-predicted muscle excitations and activations for iliacus were of similar accuracy (not shown).

Best SynX methodological choices to analyze experimental walking trials *not* used in the calibration process or to generate computationally predicted walking motions was a combination of speed-specific unmeasured synergy vector weights speed-specific residual vector weights and with 6 synergies (Figure 2). From “Params + SynX + Res” calibrations with this method, estimated muscle excitations for “iliopsoas” (left two columns) were correlated with the results from “Params” calibration strongly for left legs of both subjects and moderately for right legs of both subjects (Figure 8). There was strong correlations between muscle activations for “psoas” (right two columns) from “Params + SynX + Res” calibrations and the ones from “Params” calibrations for both legs of S1 and left leg of S2, while the correlation was moderate for right leg of S2. As for magnitude, the highest errors (RMSE = 0.1) occurred in the muscle activations for right leg of S2.

**FIGURE 9:**
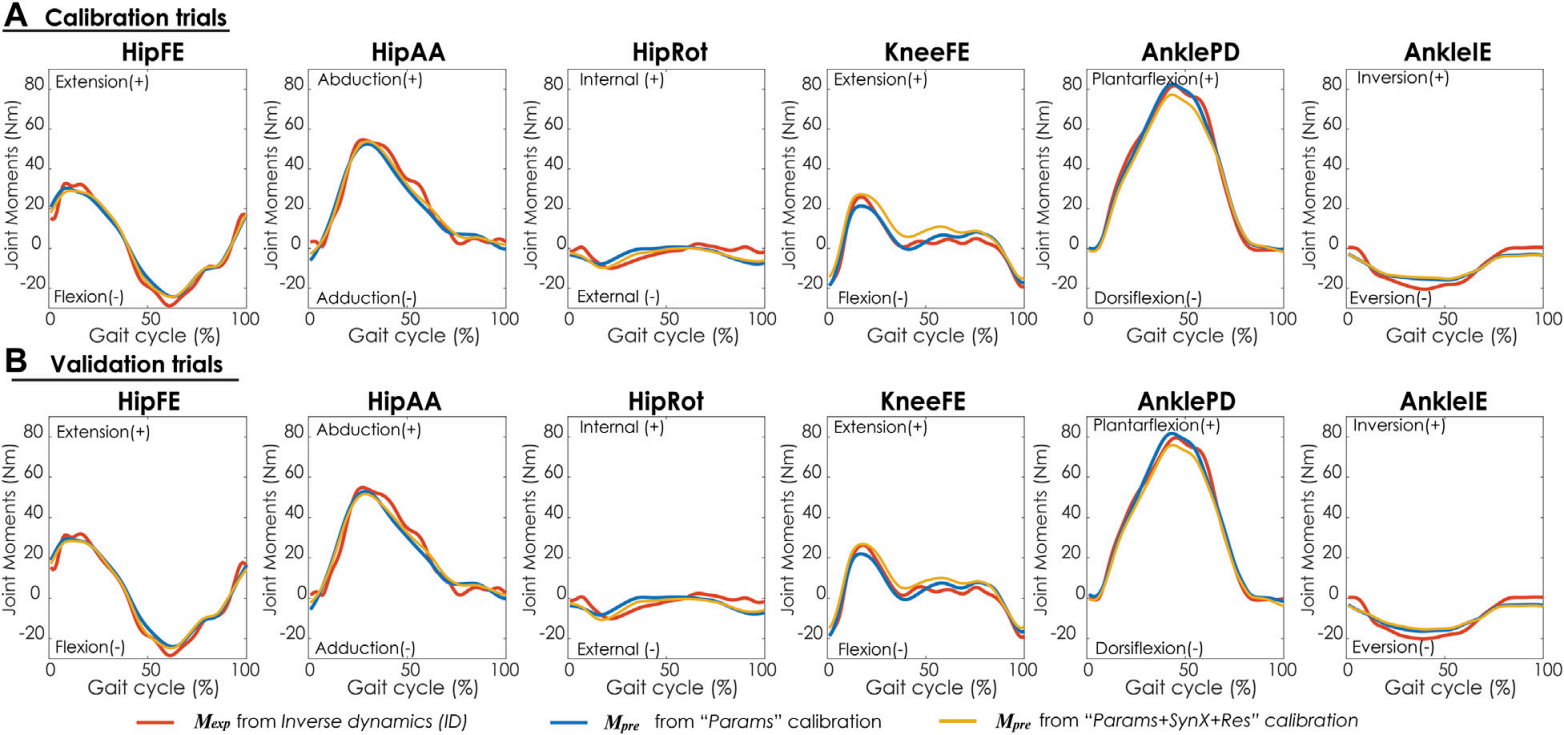
Average joint moments across trials and subjects for **(A)** calibration trials and **(B)** validation trials from inverse dynamics (in red), “Params” calibration (in blue), and “Params + SynX + Res” calibration (in yellow). Results for “Params + SynX + Res” calibration were produced using the best methodological combinations for generating computationally predicted walking motions (speed-specific unmeasured and residual synergy vector weights with six synergies). Here, residual excitations were calibrated but not used to calculate joint moments for “Params + SynX + Res.” Data are reported for the complete gait cycle, where 0% indicates initial heel-strike and 100% indicates subsequent heel-strike.

“Params + SynX + Res” calibrations with this methodological combination were able to capture experimental joint moments with reasonable accuracy (Table 4 and Figure 9A). With respect to the range of variation assumed by the average experimental joint moments, average MAE values were 0.09 for HipFE, 0.10 for HipAA, 0.22 for HipRot, 0.11 for KneeFE, 0.07 for AnklePD and 0.21 for AnkleIE. Moreover, calibrated EMG-driven models and speed-specific unmeasured synergy vector weights were capable of predicting prediction joint moments for validation trials with comparable accuracy (Table 4 and Figure 9B). Compared with the joint moments estimated from “Params”, “Params + SynX + Res” calibrations matched the ID joint moments slightly better at three hip DOFs and slightly worse at knee DOF and two ankle DOFs for both calibration trials and validation trials, whereas the difference were not statistically significant (*p* = 0.092, Table 4 and Figure 9), Additionally, for both calibration and validation trials, “Params + SynX + Res” calibration using this methodological combination could provide similar estimates of muscle activations (Supplementary Figure S5) or muscle forces (Supplementary Figure S6) to the results predicted from “Params” calibration case in terms of both shape and magnitude.

**TABLE 4:** Mean absolute error (MAE) values calculated between joint moments found from inverse dynamics and either “Params” calibration or “Params + SynX + Res” calibration.

Joint	Calibration case	Calibration				Validation			
		S1		S2		S1		S2	
		Left	Right	Left	Right	Left	Right	Left	Right
HipFE	Params	7.14	6.82	5.75	6.14	6.68	6.37	5.92	6.30
	Params + SynX + Res	6.17	5.72	5.46	5.91	6.00	6.63	5.65	5.26
HipAA	Params	6.88	7.24	7.46	5.55	6.31	7.62	7.50	6.35
	Params + SynX + Res	5.91	6.05	5.20	4.41	5.53	7.05	7.20	5.28
HipRot	Params	5.97	5.71	2.62	2.00	5.25	6.17	3.40	1.88
	Params + SynX + Res	4.79	4.25	2.40	1.96	4.25	5.69	3.27	2.12
KneeFE	Params	6.01	4.06	5.03	4.81	5.88	4.82	5.56	4.35
	Params + SynX + Res	6.54	4.40	5.77	5.15	6.69	5.08	5.52	5.35
AnklePD	Params	7.77	6.04	5.42	4.98	7.78	6.10	5.53	5.29
	Params + SynX + Res	8.11	6.30	5.60	5.26	7.08	6.27	6.37	5.33
AnkleIE	Params	4.80	2.83	7.11	1.84	4.16	2.29	6.60	2.01
	Params + SynX + Res	5.11	3.34	7.12	1.94	4.71	3.41	7.02	2.21

The results for “Params + SynX + Res” were produced using the best methodological combinations for validation (speed-specific unmeasured synergy vector weights with 6 synergies). MAE values reported for “*Params + SynX + Res”* were derived when unmeasured synergy vectors weights and average values were fixed at their calibrated values.

## 4 Discussion

In this paper, a novel computational approach was presented to address the problem of a small number of missing EMG signals during EMG-driven model calibration (Figure 1). SynX was employed to predict unmeasured muscle excitations by linearly combining synergy excitations extracted from measured muscle excitations, where the contribution of each synergy excitations to an unmeasured muscle excitation was determined by a set of unmeasured synergy vector weights. Meanwhile, residual muscle excitations constructed as a linear combination of measured synergy excitations were added to measured muscle excitations. We explored the influence of several important SynX methodological choices on prediction accuracy of unmeasured muscle excitations and muscle activations. We then identified two SynX methodological combinations to analyze experimental walking trials used in the calibration process and *not* used in the calibration process. First, a combination of trial-specific unmeasured synergy vector weights, speed-specific residual synergy vector weights and six synergies should be hired for analyzing experimental walking motions included in the calibration process. This methodological combination can not only provide accurate and reliable missing muscle excitations and activations, but estimates of muscle forces and joint moments that stayed close to the ones obtained from “Params” calibration for both subjects. Second, with a combination of speed-specific unmeasured synergy vector weights, speed-specific residual synergy vector weights and six synergies, we were still capable of estimating these important biomechanical quantities with reasonable accuracy. More importantly, these methods permitted us to analyze experimental walking trials not used in the calibration process or generate computationally predicted walking motions.

Synergy extrapolation (SynX) has been an emerging approach to predict unmeasured muscle excitations by the use of muscle synergy information extracted from measured muscle excitations (Bianco et al., 2018; Ao et al., 2020). Bianco et al. investigated the theoretical feasibility of using synergy excitations extracted from a group of eight “included” muscle excitations treated as measured to construct muscle excitations for a group of eight “excluded” muscle excitations treated as unmeasured (Bianco et al., 2018). To take a step forward, a previous paper from the author has shown that SynX could provide accurate estimates of unmeasured muscle excitations, where unmeasured synergy vector weights were identified through non-linear optimizations by incorporating a well-calibrated EMG-driven model (Ao et al., 2020). However, the “well-calibrated” EMG-driven model was obtained with the knowledge of muscle excitations being treated as unmeasured. Thus, this paper had achieved to implement SynX without demands of EMG data that were sought to be predict, which marked the feasibility of SynX in practical applications. Moreover, EMG-driven model calibration with SynX was formulated such that both unmeasured muscle excitations and EMG-driven model parameters were defined simultaneously, which provided advantages for both EMG-driven model personalization and missing muscle excitation prediction.

Calibration of synergy-structured residual muscle excitations were included in the proposed framework to improve the accuracy of SynX-predicted unmeasured muscle excitations (Figure 1). Noted that even though we performed a sequence of steps to improve model correctness and subject-specificity prior to EMG-driven model calibration, there were still some resources of errors that affected accuracy of joint moment estimation, such as marker location errors, soft tissue movement errors, and surface EMG measurement errors (Lloyd and Besier, 2003; Manal and Buchanan, 2013; Meyer et al., 2017). Therefore, when unmeasured muscle excitations became design variables that were iteratively adjusted within EMG-driven model calibration for SynX, they inclined to deviate from experimental muscle excitations such that joint moment matching errors were minimized (see “Params + SynX” calibrated results in Figure 2 and Supplementary Figure S2 which were labeled as “None” for the category of residual synergy vector weights). Residual muscle excitations were introduced to account for joint moment matching errors, which facilitated to prevent predicted missing muscle excitations from excessively compensating for joint moment prediction inaccuracy through optimization and becoming inaccurate as a consequence.

Even though residual muscle excitations were needed to predict within “Params + SynX + Res” optimization, our EMG-driven model calibration method could still generate two sets of joint moments, depending on whether residual muscle excitations were included or not for joint moment calculation. To illustrate the discrepancy in results between “Params + SynX + Res” and “Params” calibrations, we consistently presented the muscle activations (Figure 5), muscle forces (Figure 6) and joint moments (Figure 4) calculated without inclusion of residual muscle excitations. However, joint moment prediction errors could be substantially reduced (by averagely 3 Nm) when including residual muscle excitations for joint moment calculation (Supplementary Table S2). Thus, joint moments outputted from our framework could be highly dependent on the actual demands, where residual muscle excitations could be included in the prediction of joint moments, if the difference between model-predicted and experimental joint moments are required to be as low as possible.

As a follow-up step of “Params” calibration, we calibrated the residual excitations needed for all the muscles (including psoas and iliacus) to match joint moments from inverse dynamics better (termed “Res” calibration), where the EMG-driven model parameters were held constant with the values obtained through “Params” calibration. We found that joint moment tracking accuracy was significantly improved by including synergy-structured residual excitations (*p* < 0.05), where the extent of improvements were dependent on the category of residual synergy vector weights (Supplementary Figure S3). More interestingly, we observed that within the regions of gait cycle where SynX-predicted muscle excitations and activations from “Params + SynX + Res” calibration could not match well with the ones from “Params” calibration, they showed good agreement with the ones from “Res” calibrations (Supplementary Figures S8, S9). Therefore, we believed that SynX-predicted muscle excitations and activations within our framework were likely superposition of “experimental” muscle activities and corresponding synergy-structured residual muscle activities needed for psoas and iliacus to improve joint moment matching.

For several important reasons, synergy structures were imposed on both unmeasured muscle excitations and residual muscle excitations in our framework. First, unlike SO that solved a time frame of muscle activation at a time (Anderson and Pandy, 2001; Ackermann and van den Bogert, 2010; Sartori et al., 2014; Zonnino and Sergi, 2019), unmeasured and residual synergy vector weights were time-invariant, which opened the possibility of a single-layer optimization process to simultaneously achieve EMG-driven model personalization and missing muscle excitation prediction. Second, muscle excitation-activation relationships were defined by a set of differential equations (Meyer et al., 2017), synergy-structured excitations could be resolved over all time frames at a time, which allowed us to perform reconstruction at level of muscle excitations and thus to decrease the search space for the optimization in comparison with SO-based approaches. Third, the problem of finding unknown time-varying activations was reduced to a problem of finding a smaller number of unmeasured and residual synergy vector weights, which significantly decreased the search space for the optimization in comparison with SO-based approaches. Fourth, due to inherent constraints of dependence between time frames, synergy-structured residual excitations showed predictably worse joint moment matching performance than did SO-based residual activations (Supplementary Figure S3). However, they could still lower joint moment matching errors by significant amount, especially when using trial-specific synergy vector weights (Supplementary Figure S3). When both unmeasured muscle excitations and residual excitations were consistently constructed using synergy concepts, the joint moment matching errors that resultant unmeasured muscle excitations could potentially over-compensate for would be sufficiently accounted for by the resultant residual excitations.

An optimization problem was formulated within our EMG-driven modeling method that minimized four primary cost terms (Eq. 15) simultaneously, where a combination of maximum allowable deviation (MAD) values needed for normalization of cost values was determined through a series of sensitivity tests such that satisfactory SynX outcomes were consistently generated across legs of both subjects (Supplementary Figure S1). On the one hand, joint moment tracking errors were minimized when residual muscle excitations were (Eq. 16) and were not (Eq. 17) used for joint moment calculation. Both terms were included such that residual excitations for measured muscles were estimated with reasonable accuracy and EMG-driven model parameters for measured muscles stayed as close as possible to the ones derived from “Params” calibration. On the other hand, unmeasured and residual muscle activations were minimized such that the optimization did not incline to converge to a set of overestimated solutions. Here, we implemented minimization at the activation-level instead of excitation-level, because muscle activations were more consistent in terms of magnitudes across muscles and subjects, which facilitated finding a consistent set of maximum allowable deviation values (MAD) that could provide acceptable outcomes across legs of both subjects.

There were a number of methodological choices required to make during muscle synergy analysis (MSA) that could influence the results of extracted measured synergy excitations and corresponding synergy extrapolation performance, such as EMG normalization methods, number of synergies, matrix decomposition algorithm (Ivanenko et al., 2005; Tresch et al., 2006; Steele et al., 2013; Oliveira et al., 2014; Banks et al., 2017; Shuman et al., 2017; Ebied et al., 2018; Gallina et al., 2018; Ao et al., 2020) In a previous study (Ao et al., 2020), the author reported that PCA provided more accurate, reliable, and efficient estimates of unmeasured muscle excitations than NMF when using a well-calibrated EMG-driven model. A similar observation was made in this study that when the number of synergies and categories of unmeasured and residual synergy vector weights were matched, NMF could not produce estimates of unmeasured muscle excitations and activations from “Params” calibration as accurately as was PCA (Supplementary Figure S7). As highlighted in the previous paper, the reasons PCA outperformed NMF for SynX could be due to nonnegativity constraints for NMF and extra design variables for PCA, both of which could make the feasible search space of NMF more restricted than that of PCA. Beyond these, PCA was especially advantageous in our framework because it allowed residual excitations to be both positive and negative, which could be beneficial to achievement of lower joint moment errors.

The principal methodological decisions explored in this study were the number of synergies and variability of synergy vector weights across trials. With an increasing number of synergies, SynX performance exhibited non-monotonic behavior for both subjects, where six synergies provided the generally best SynX performance and EMG-driven model calibration outcomes for both legs of S1 and left leg of S2 and five synergies provided the best overall results for right leg of S2. The results were in great agreement with the findings reported in (Ao et al., 2020). Additionally, based on the assumptions about the variability of synergy vector weight across walking trials, we categorized them associated with unmeasured and residual muscle excitations as trial-specific, speed-specific, and subject-specific, respectively, while different concatenation strategies were used to extract corresponding synergy excitations. We evaluated the results with all possible methodological combinations (Figure 2 and Supplementary Figure S2), and it was indicated that with matched number of synergies, trial-specific unmeasured synergy vector weights and speed-specific residual synergy vector weights generated the best SynX performance for most legs. For both measured (Table 3) and unmeasured (Figure 2 and Supplementary Figure S2) muscle excitations, a small number of synergies (e.g., <5) or subject-specific synergy vector weights may not be sufficient to account for their variance. As the number of synergies or the variability of synergy vector weights across trials increased, the additional degrees of freedom in the optimization allowed the optimizer to lower the joint moment matching errors (Supplementary Figure S4). However, when the flexibility determined by the number of synergies and category of synergy vector weights was above a certain level, the joint moment tracking errors dropped below those achieved by calibration with a complete set of EMG data (“Params” calibration), where the prediction of unmeasured muscle excitations and activations becoming less accurate as a consequence. Another important merit of using speed-specific residual synergy vector weights was that computational cost would be drastically reduced because we normally have way more channels of measured EMGs than channels of unmeasured EMGs when using our framework.

Even though we suggested employing six synergies, which provided the most accurate predictions for most legs, the overall SynX and model calibration performance peaked at five synergies for the right leg of S2 (Figure 2 and Supplementary Figure S2). The underlying reason for this inconsistency could be that subject S2 was a low-functioning post-stroke subject, whose right leg was paretic. Clark et al. reported that post-stroke subjects had fewer muscle synergies which could result from the merging of the synergies observed in healthy controls due to impaired locomotor coordination and the reduced independence of neural control signals (Clark et al., 2009). We made a consistent observation within our MSA results that the right leg of S2 had generally higher VAF values when the number of synergies and category of synergy vector weights were matched. Also, when assuming synergy vector weights speed-specific and subject-specific, the right leg of S2 required four or five synergies to account for over 95% of the variability in measured muscle excitations, while the left leg of S2 and both legs of S1 needed six or seven synergies. Therefore, it is reasonable to speculate that for the paretic leg of S2, five synergies were sufficient for SynX when hiring our framework, while six synergies could introduce additional degrees of freedom to the optimization problem and influence the SynX performance adversely.

This study possessed several limitations that could help inform future research efforts. First, this study validated the performance of our framework using gait datasets from two subjects post-stroke who had a complete set of EMG data from muscles in the lower extremities. Broader investigations need to be done in diverse subject populations of larger sample sizes. Second, our framework was validated in cases where EMG data from only one important hip muscle group (iliopsoas) was assumed to be missing at a time. The approach should be evaluated more extensively in cases where more important deep muscles are assumed to be unmeasured individually or concurrently. Third, we developed the framework using walking data with two representative speeds. We are planning to explore the framework’s feasibility for other types of dynamic movement conditions and experimental scenarios, such as stair climbing and running. Lastly, to achieve the purpose of computational prediction for the trials withheld from calibration, we suggested assuming unmeasured and residual synergy vector weights speed-specific for walking. When our framework is applied for diverse motion tasks, it is worthwhile exploring the feasibility of making a hybrid assumption about the variability of synergy vector weights, such as speed-specific synergy vector weights mixed with task-specific synergy vector weights.

## 5 Conclusion

In conclusion, we have presented a novel computational method that can address the problem of a small number of missing EMG signals during EMG-driven model calibration. Within this method, synergy excitations extracted from measured muscle excitations using PCA were linearly combined to reconstruct unmeasured muscle excitations (termed “synergy extrapolation” or “SynX”) and residual muscle excitations for measured muscles. The unmeasured and residual synergy vector weights defining the contribution of each measured synergy excitation to all muscle excitations were identified simultaneously with EMG-driven model parameters through a multi-objective optimization. The study also assessed how SynX methodological choices (i.e., number of synergies and category of unmeasured synergy vector weights and residual synergy vector weights) influenced SynX performance. By comparing with results from EMG-driven model calibration using a complete set of EMG signals, we identified two SynX methodological combinations for the purposes of analyzing experimental walking trials used in the calibration process and *not* used in the calibration process, respectively. Both methodological combinations consistently provided accurate, reliable, and efficient estimates of both SynX-relevant quantities (i.e., missing muscle excitations and activations) and biomechanical variables (i.e., muscle forces and joint moments). This computational approach opens up possibilities for the personalization of EMG-driven musculoskeletal models when difficulties exist with collecting EMG signals from important muscles.

## Data Availability

The experimental data, OpenSim model, and Matlab code used to perform this study are available at https://simtk.org/projects/synx.
