# Supplementary material for: EMG-driven musculoskeletal model calibration with estimation of unmeasured muscle excitations via synergy extrapolation
Source: Front Bioeng Biotechnol. 2022 Sep 7;10:962959. doi: 10.3389/fbioe.2022.962959 (PMC9490010; doi:10.3389/fbioe.2022.962959)
Supplement: Supplementary file 1 [file DataSheet1.docx]

Supplementary Material

Table S1: Initial guesses, upper bounds and lower bounds for each design variable for calibration

| Symbol | Description | Initial guess | Lower bounds | Upper bounds |
| --- | --- | --- | --- | --- |
| $d$ | electromechanical delay | 0.5 | 0 | 1.25 |
| $\tau_{act}$ | activation time constant | 1.5 | 0.6 | 3.5 |
| $c_{3}$ | activation nonlinearity constant | 0.05 | 0 | 0.35 |
| $s_{EMG}$ | EMG scale factors | 0.5 | 0.05 | 1 |
| $l_{o}^{M}$ | optimal muscle fiber length | 1 | 0.6 | 1.4 |
| $l_{s}^{T}$ | tendon slack length | 1 | 0.6 | 1.4 |
| $H_{SynX}$ | unmeasured synergy weights | 0 | -Inf | Inf |
| $\mu_{SynX}$ | mean unmeasured excitations | 0 | -Inf | Inf |
| $H_{res}$ | residual synergy weights | 0 | -Inf | Inf |
| $\mu_{res}$ | mean residual excitations | 0 | -Inf | Inf |

## Maximum allowable errors in cost function terms

A series of sensitivity tests were performed to identify a combination of maximum allowable deviation values (${MAD}_{1-4}$) that ensured unmeasured muscle excitations were consistently predicted with reasonable accuracy across both legs of both subjects. ${MAD}_{1}$ for minimizing joint moment matching errors with residual excitations applied was fixed at 2 Nm to be consistent with our previous studies (Meyer et al., 2017) (Ao et al., 2020). ${MAD}_{2}$ for minimizing joint moment matching errors without residual excitations applied was chosen to be 2, 2.5 or 3 Nm, while ${MAD}_{3}$ for minimizing unmeasured muscle activations and ${MAD}_{4}$ for minimizing residual muscle activations were chosen to be between 0.1 and 0.9 with an increment of 0.2. We performed 75 ($5\times5\times3$) repetitive analyses with each possible combination of $MAD$ values while using 5 PCA-based synergy excitations extracted on a trial-by-trial basis to reconstruct unmeasured muscle excitations. We used 5 PCA-based synergy excitations extracted on a speed-by-speed basis to reduce computation time to reconstruct residual muscle excitations. To focus on the primary terms associated with SynX during this sensitivity investigation, we fixed activation dynamics model parameters and muscle-tendon model parameters to the values produced by full EMG-driven calibration. r values and RMSE values were calculated for muscle excitations and muscle activations between “Params+SynX+Res” calibration case and “Params” calibration case to quantify the SynX performance. As shown in figure 1, when SynX performance for both subjects was considered as a whole, a combination of ${MAD}_{1} = 2$, ${MAD}_{2} = 2.5$, ${MAD}_{3} = 0.5$, and ${MAD}_{4} = 0.5$ could provide the best reconstruction of unmeasured muscle excitations and activations through “ Params+SynX+Res” calibrations in magnitude and shape.


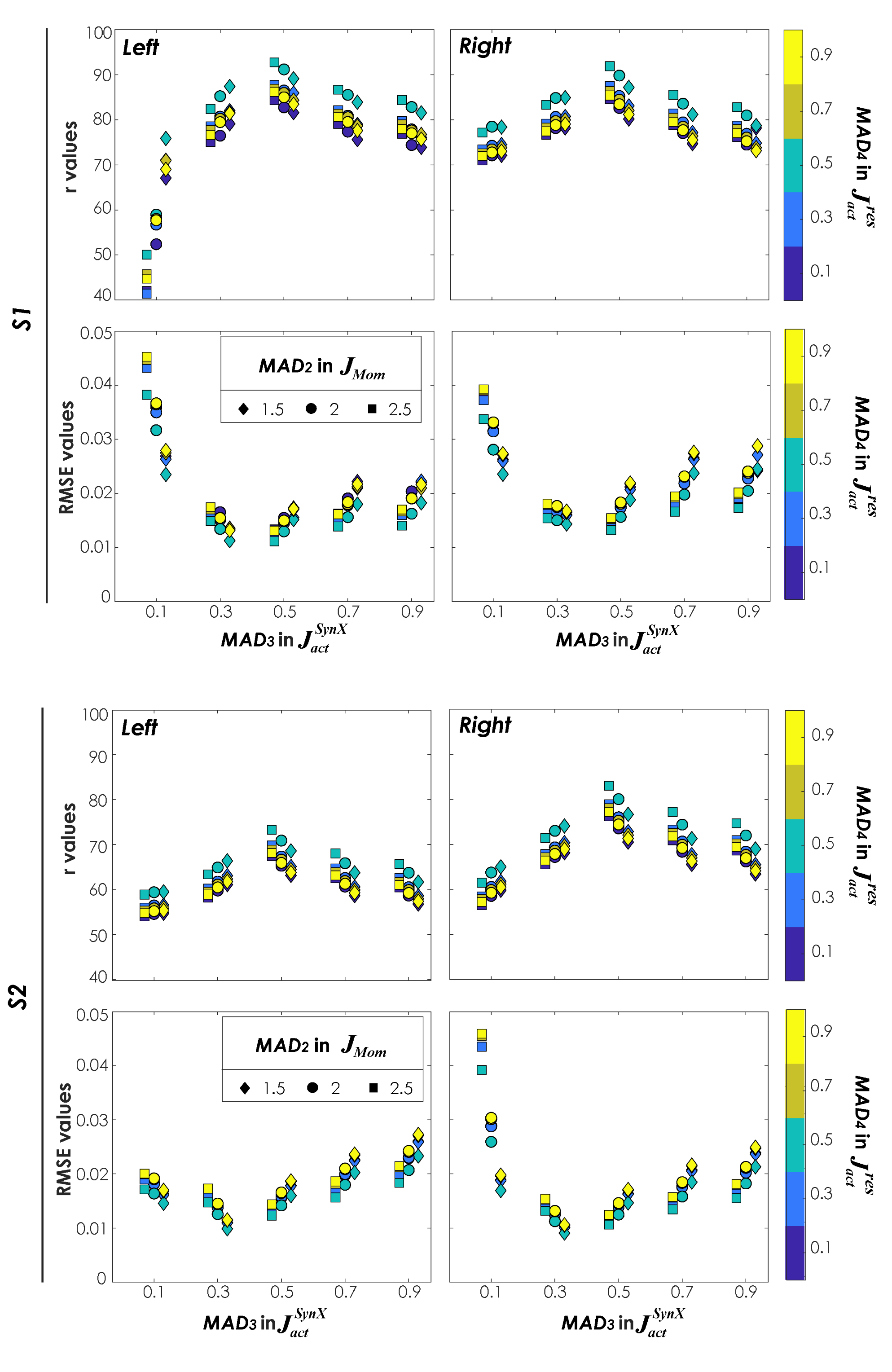


Figure S1 r values and RMSE values for muscle activations of psoas calculated from between “Params+SynX+Res” calibration case and “Params” calibration case for each combination of maximum allowable errors in the cost function. The sensitivity test was performed with 5 synergies to reconstruct unmeasured muscle excitations and residual excitations. All EMG-driven model parameters were given by the results from “Params” calibration case.


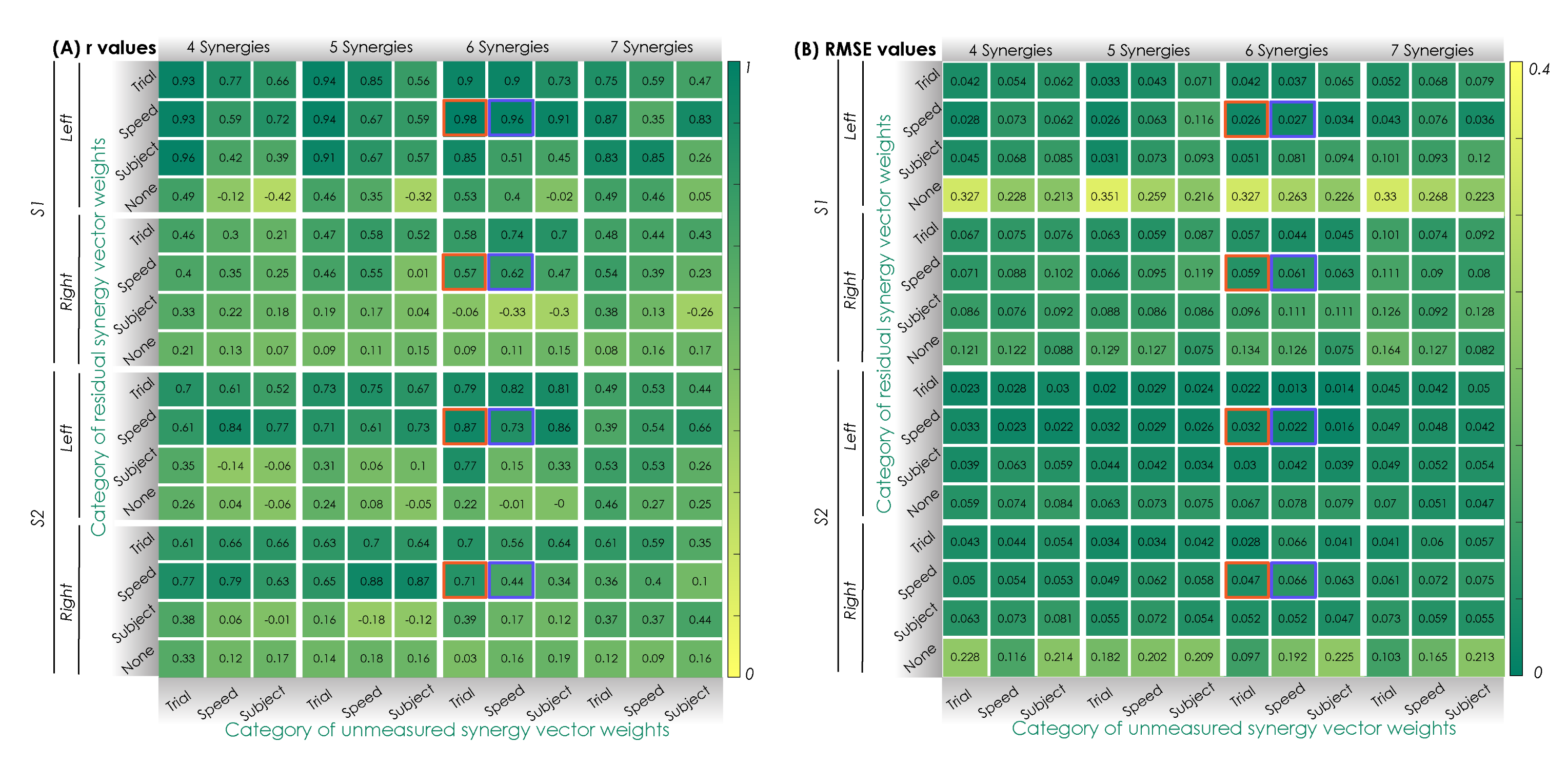


Figure S2: Synergy extrapolation performance for different methodological combinations when using the proposed EMG-driven calibration framework. (A) Pearson correlation coefficient r values and (B) root mean square error (RMSE) values for different methods of reconstructing psoas muscle excitations across all calibration trials. Unmeasured (bottom) and residual (side) synergy vector weights were categorized as either trial-specific, speed-specific, or subject-specific. Within the columns for 6 synergies, Orange boxes indicate the best SynX methodological combination (trial-specific unmeasured and speed-specific residual synergy vector weights) for analyzing an experimentally measured walking motion. The purple boxes indicate the best SynX methodological combination (speed-specific unmeasured and speed-specific residual synergy vector weights) for generating a computationally predicted walking motion. Residual synergy vector weights categorized as “None” indicate calibration results for “Params+SynX,” where no residual muscle excitations were predicted. These results suggest that “Params+SynX” calibration should be rejected due to unacceptable SynX performance.

Table S2 Mean absolute error (MAE) values calculated for joint moments between inverse dynamics and “Params” calibration case or between inverse dynamics and “Params+SynX+Res” calibration case, respectively. The results for “Params+SynX+Res” calibrations were generated with the best performing methodological combinations for calibration (trial-specific unmeasured and speed-specific residual synergy vector weights with 6 synergies).

| Joints | Calibration case | Residual excitations | S1 | | S2 | | |
| --- | --- | --- | --- | --- | --- | --- | --- |
|  |  |  | Left | Right | Left | Right |  |
| HipFE | Params |  | 7.14 | 6.82 | 5.75 | 6.14 | |
|  | Params+SynX+Res | *w/o* | 6.42 | 6.41 | 5.43 | 5.70 | |
|  |  | *w/* | 3.21 | 2.28 | 2.39 | 2.63 | |
| HipAA | Params |  | 6.88 | 7.24 | 7.46 | 5.55 | |
|  | Params+SynX+Res | *w/o* | 6.70 | 7.19 | 7.26 | 5.79 | |
|  |  | *w/* | 4.62 | 4.22 | 4.09 | 3.19 | |
| HipRot | Params |  | 5.97 | 5.71 | 2.62 | 2.00 | |
|  | Params+SynX+Res | *w/o* | 5.51 | 5.49 | 2.36 | 2.03 | |
|  |  | *w/* | 3.88 | 2.65 | 1.90 | 1.49 | |
| KneeFE | Params |  | 6.01 | 4.06 | 5.03 | 4.81 | |
|  | Params+SynX+Res | *w/o* | 5.81 | 3.91 | 4.90 | 4.70 | |
|  |  | *w/* | 3.32 | 2.37 | 2.35 | 2.19 | |
| AnklePD | Params |  | 7.77 | 6.04 | 5.42 | 4.98 | |
|  | Params+SynX+Res | *w/o* | 7.12 | 5.91 | 5.35 | 4.97 | |
|  |  | *w/* | 4.15 | 3.70 | 3.87 | 2.01 | |
| AnkleIE | Params |  | 4.80 | 2.83 | 7.11 | 1.84 | |
|  | Params+SynX+Res | *w/o* | 4.67 | 2.11 | 7.02 | 1.70 | |
|  |  | *w/* | 2.18 | 1.24 | 2.74 | 0.88 | |

*w/o :* residual excitations were calibrated but not used to calculate joint moments for “Params+SynX+Res.” *w/:* residual excitations were calibrated and used to calculate joint moments for “Params+SynX+Res.”


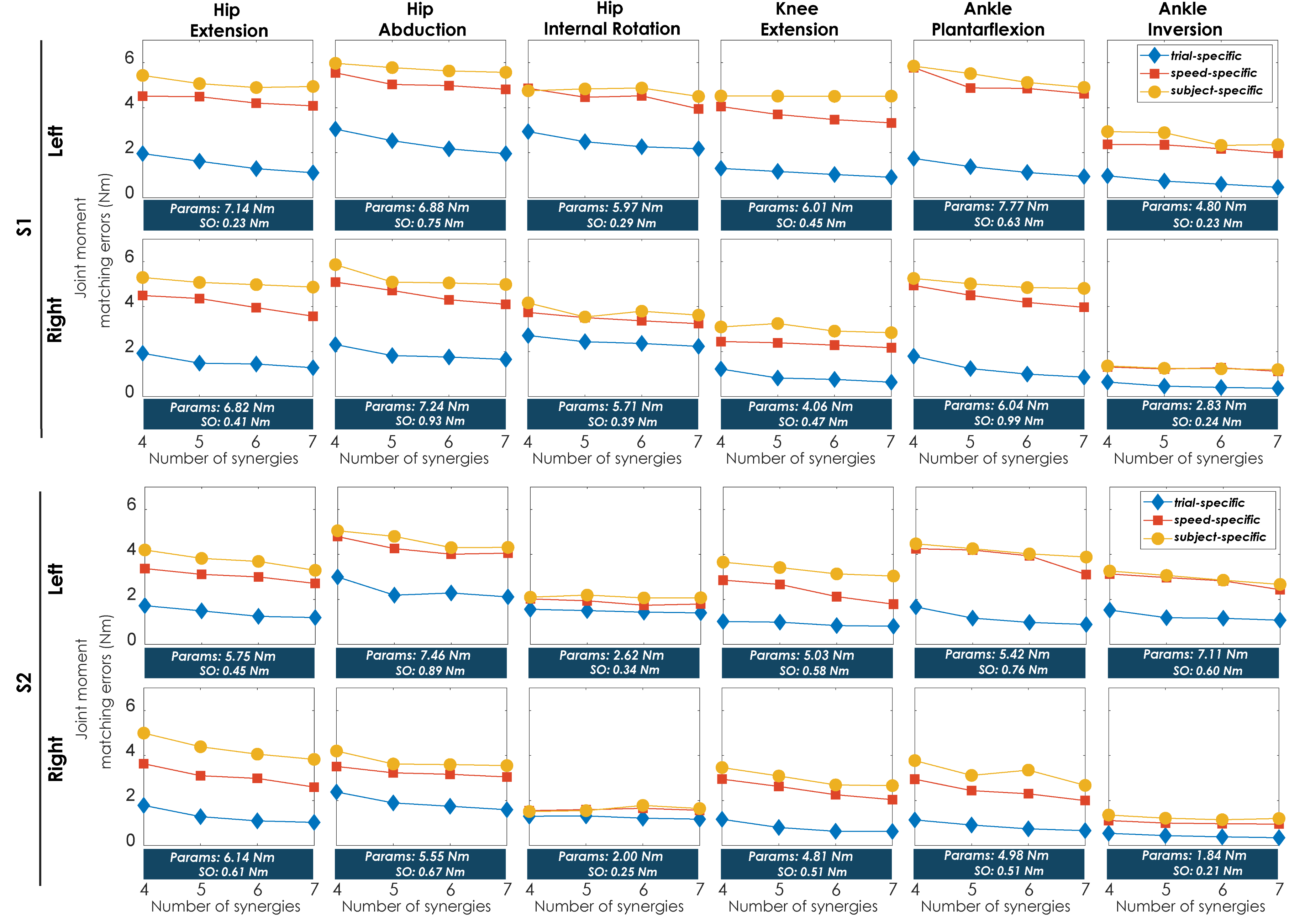


Figure S3: Mean absolute errors (MAEs) of joint moment tracking when performing “Res” calibration with different methodological combinations. During these process, all EMG-driven model parameters were given by the results from “Params” calibration case. Residual synergy weights were assumed to be trial-specific, speed-specific, and subject-specific, respectively. In the box below each subplot, “Params” indicated the joint moment tracking error value from “Params” calibration, and “SO” indicated joint moment tracking error value when residual activations applied to all muscles were estimated with the static optimization-based approach.


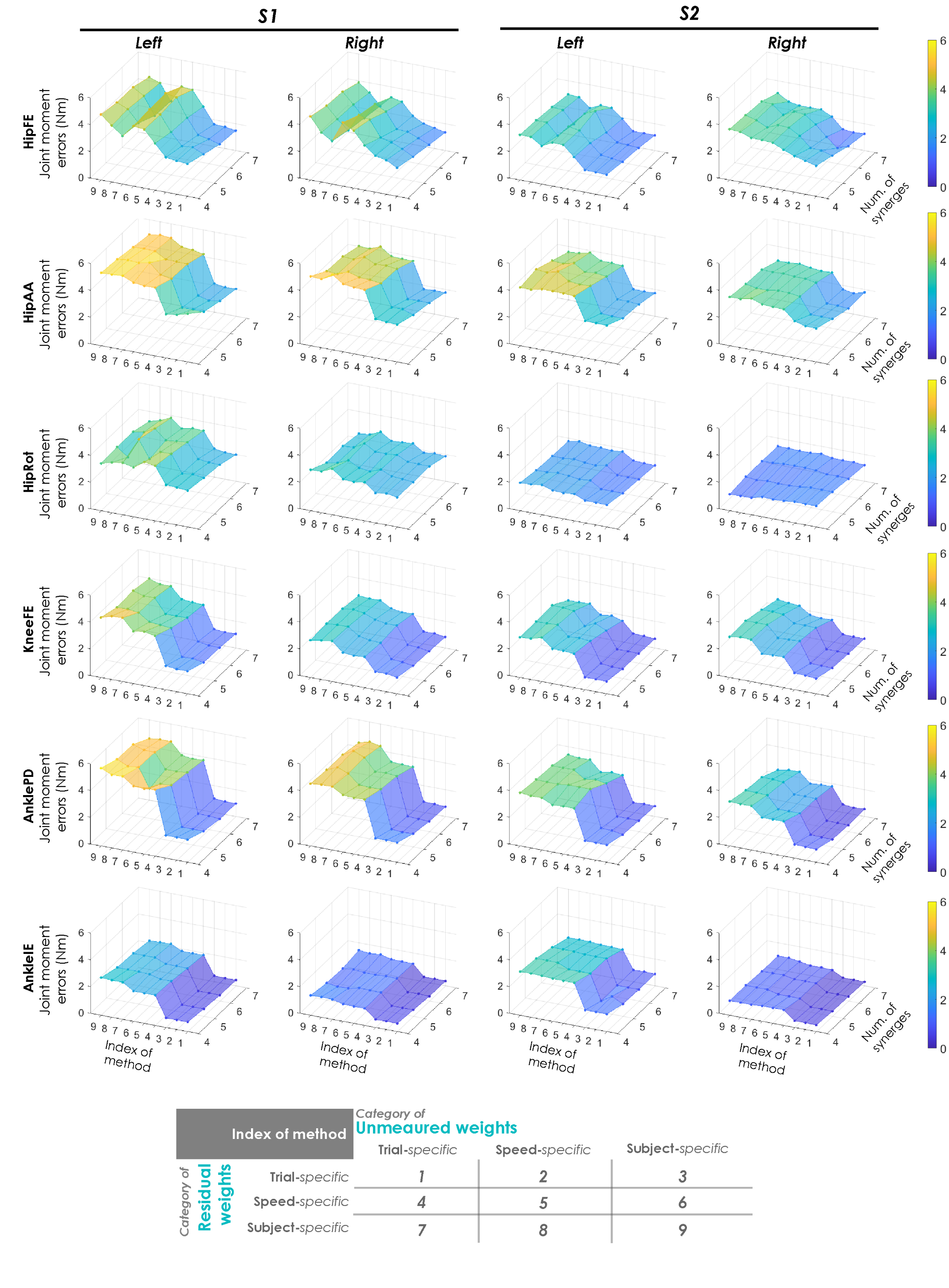


Figure S4: Mean absolute error (MAE) values calculated for joint moments between inverse dynamics and “Params+SynX+Res” calibration case. Here, residual excitations were calibrated and also used to calculate joint moments for “Params+SynX+Res”.


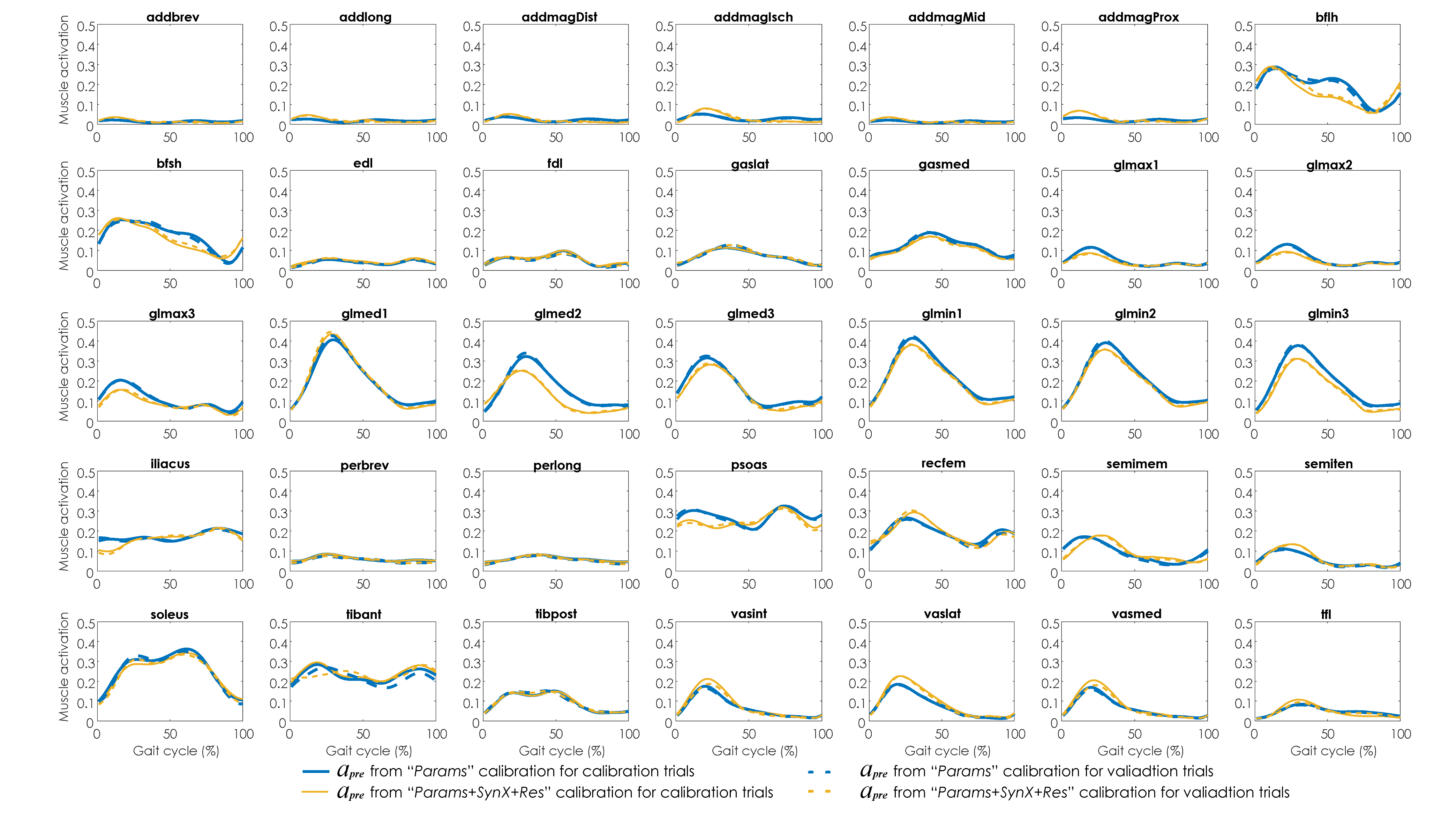


Figure S5: Average muscle activations across calibration trials (solid lines) and validation trials (dash lines) for both subjects from “Params” calibration (in blue), and “Params+SynX+Res” calibration (in yellow). Results for “Params+SynX+Res” calibration were generated using the best methodological combinations for analyzing experimentally measured walking motions (trial-specific unmeasured and speed-specific residual synergy vector weights with 6 synergies). Here, residual excitations were calibrated but not used to calculate muscle activations for “Params+SynX+Res.” Data are reported for the complete gait cycle, where 0% indicates initial heel-strike and 100% indicates subsequent heel-strike.


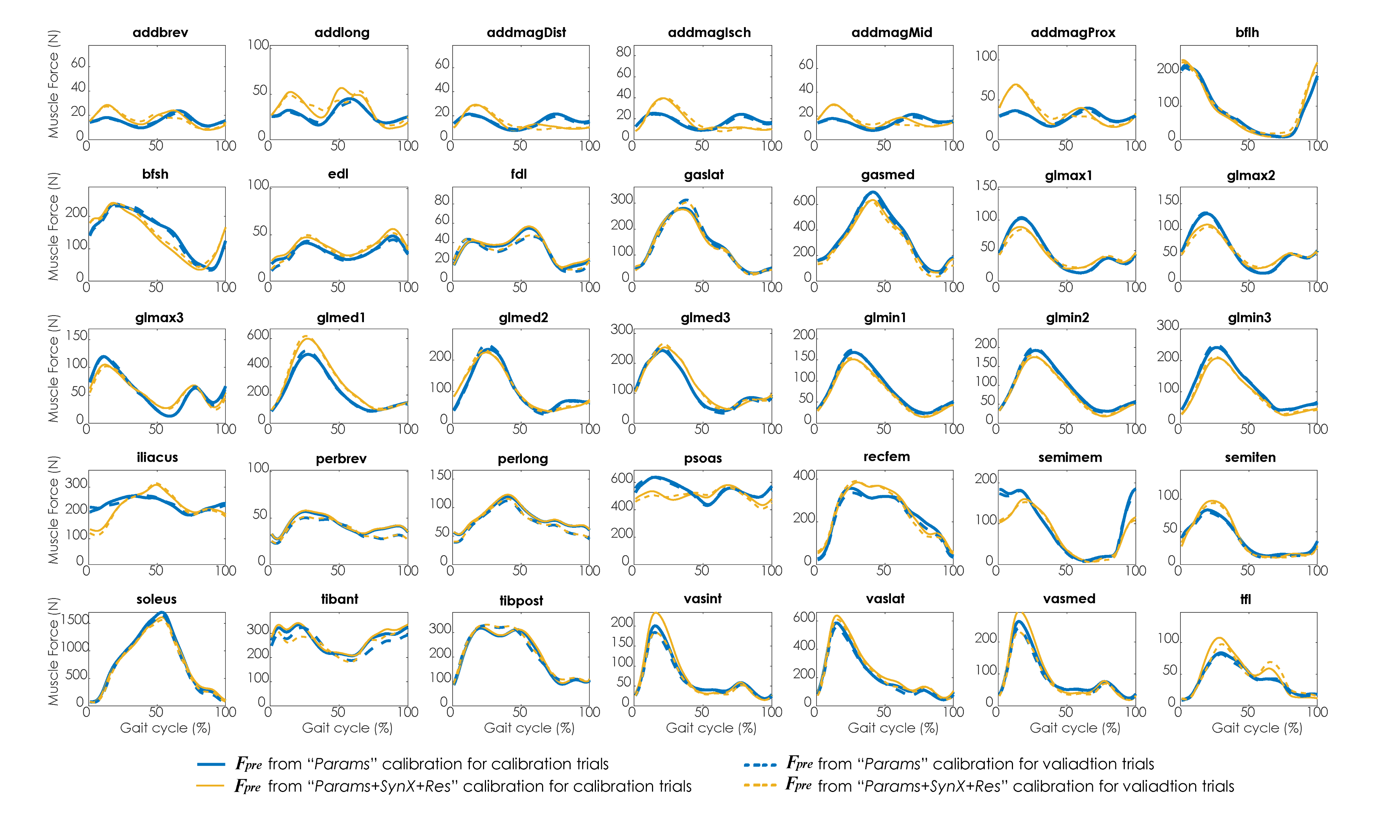


Figure S6: Average muscle forces across calibration trials (solid lines) and validation trials (dash lines) for both subjects from “Params” calibration (in blue) and “Params+SynX+Res” calibration (in yellow). Results for “Params+SynX+Res” calibration were produced using the best methodological combinations for analyzing experimentally measured walking motions (trial-specific unmeasured and speed-specific residual synergy vector weights with 6 synergies). Here, residual excitations were calibrated but not used to calculate muscle forces for “Params+SynX+Res.” Data are reported for the complete gait cycle, where 0% indicates initial heel-strike and 100% indicates subsequent heel-strike.


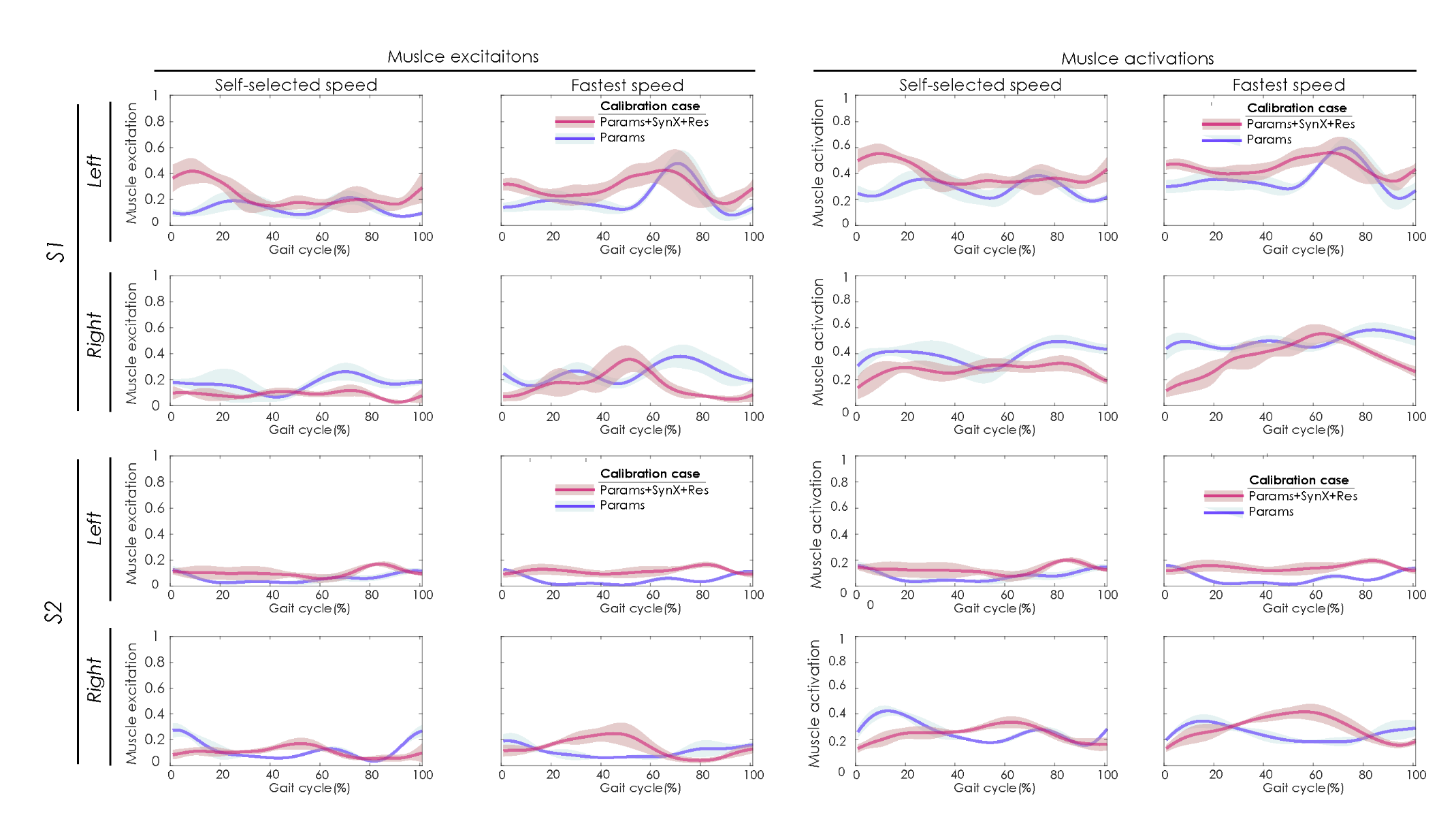


Figure S7: SynX-predicted muscle excitations and activations with non-negative matrix factorization (NMF) for psoas constructed with the best methodological combinations for analyzing experimentally measured walking motions (trial-specific unmeasured and speed-specific residual synergy vector weights with 6 synergies). Lines represent mean curves across calibration trials and shaded areas represent ±1 standard deviation. “Params” calibration was performed using a complete set of EMG signals, where no muscle excitations were predicted. Data are reported for the complete gait cycle, where 0% indicates initial heel-strike and 100% indicates subsequent heel-strike for each leg of both subjects (right leg: paretic, left leg: nonparetic). SynX-predicted muscle excitations and activations for iliacus were similar to those for psoas, thus not presented in this plot.


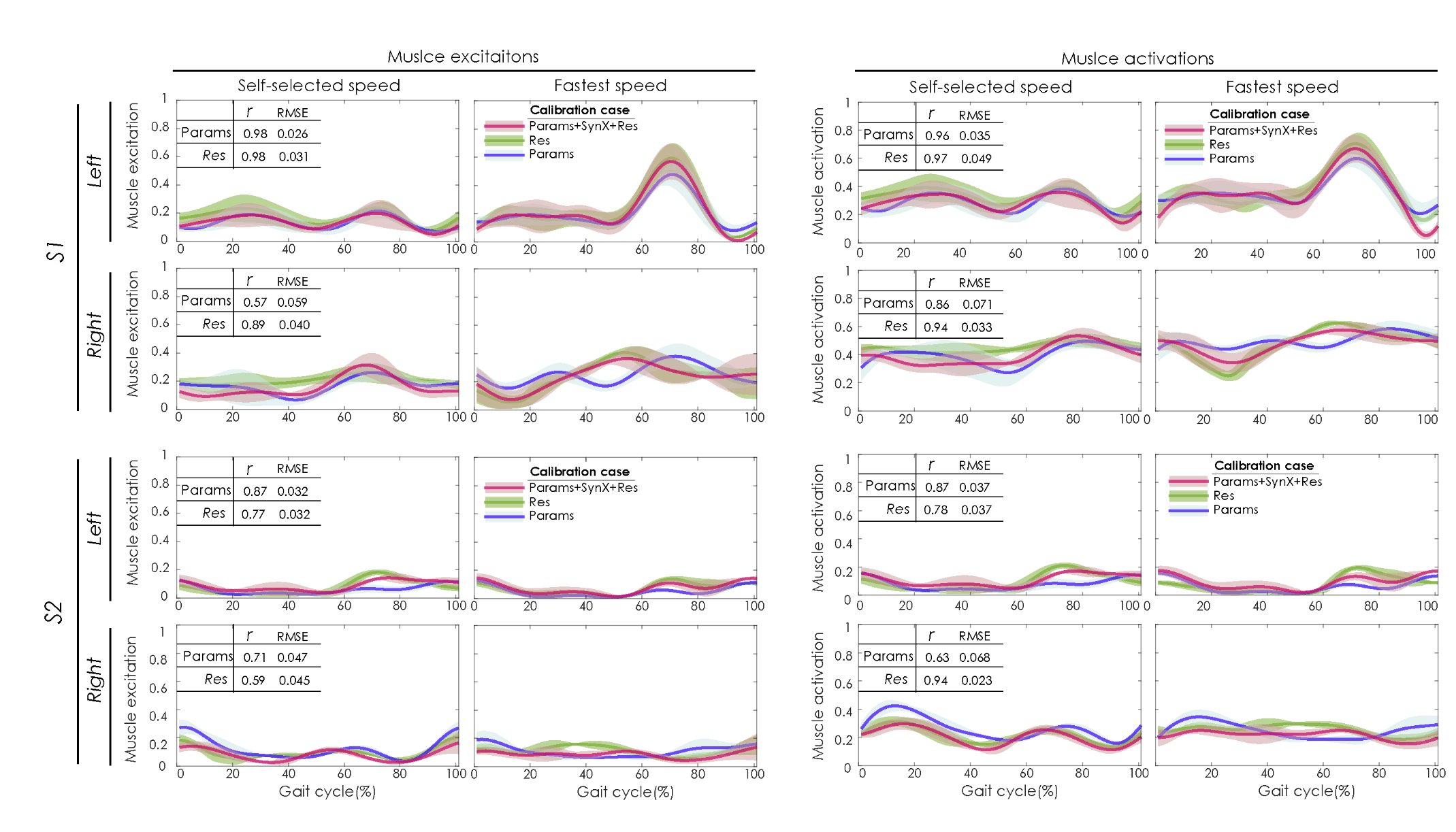


Figure S8: SynX-predicted muscle excitations and activations for psoas constructed with the best methodological combinations for analyzing experimentally measured walking motions (trial-specific unmeasured and speed-specific residual synergy vector weights with 6 synergies). Lines represent mean curves across calibration trials and shaded areas represent ±1 standard deviation. r and RMSE values for muscle excitations and activations were calculated between “Params+SynX+Res” calibration (in pink) and either “Params” calibration (in blue) or “Res” calibration (in green). “Params” calibration was performed using a complete set of EMG signals, where no muscle excitations were predicted. “Res” calibration was performed as a follow-up procedure to determine better the residual excitations needed for psoas to match joint moments from inverse dynamics. Data are reported for the complete gait cycle, where 0% indicates initial heel-strike and 100% indicates subsequent heel-strike for each leg of both subjects (right leg: paretic, left leg: nonparetic). SynX-predicted muscle excitations and activations for iliacus were similar to those for psoas, thus not presented in this plot.


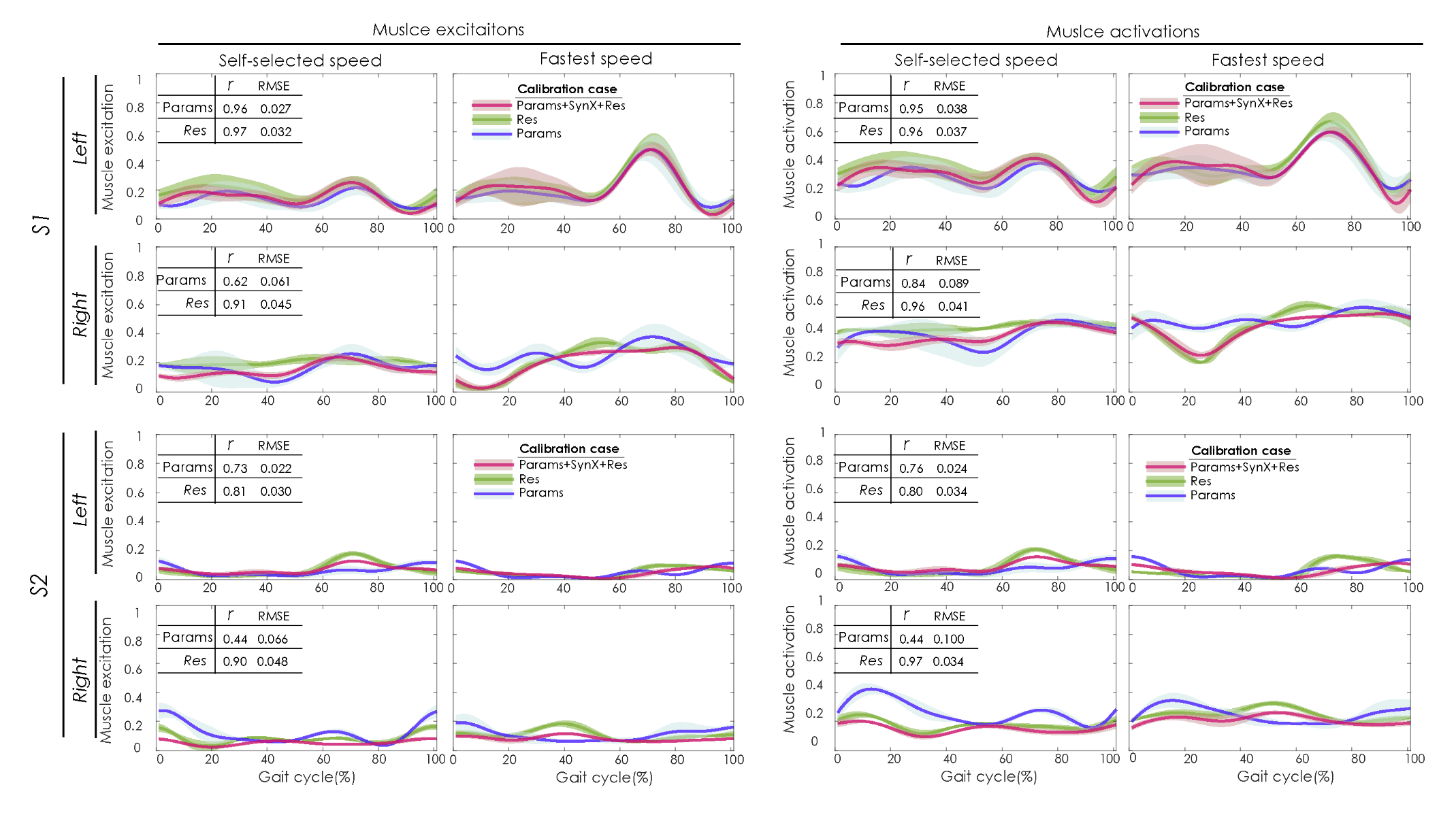


Figure S9: SynX-predicted muscle excitations and activations for psoas constructed with the best methodological combinations for generating a computationally predicted walking motion (trial-specific unmeasured and speed-specific residual synergy vector weights with 6 synergies). Lines represent mean curves across calibration trials and shaded areas represent ±1 standard deviation. r and RMSE values for muscle excitations and activations were calculated between “Params+SynX+Res” calibration (in pink) and either “Params” calibration (in blue) or “Res” calibration (in green). “Params” calibration was performed using a complete set of EMG signals, where no muscle excitations were predicted. “Res” calibration was performed as a follow-up procedure to determine better the residual excitations needed for psoas to match joint moments from inverse dynamics. Data are reported for the complete gait cycle, where 0% indicates initial heel-strike and 100% indicates subsequent heel-strike for each leg of both subjects (right leg: paretic, left leg: nonparetic). SynX-predicted muscle excitations and activations for iliacus were similar to those for psoas, thus not presented in this plot.
